# Structural Properties and Magnetic Ground States of 100 Binary *d*-Metal Oxides Studied by Hybrid Density Functional Methods

**DOI:** 10.3390/molecules27030874

**Published:** 2022-01-27

**Authors:** Mikhail S. Kuklin, Kim Eklund, Jarno Linnera, Artturi Ropponen, Nikolas Tolvanen, Antti J. Karttunen

**Affiliations:** Department of Chemistry and Materials Science, Aalto University, FI-00076 Aalto, Finland; mikhail.kuklin@aalto.fi (M.S.K.); kim.eklund@aalto.fi (K.E.); jarno.linnera@aalto.fi (J.L.); artturi.ropponen@gmail.com (A.R.); ntolvanen.com@gmail.com (N.T.)

**Keywords:** oxides, transition metals, magnetism, computational chemistry, density functional theory

## Abstract

*d*-metal oxides play a crucial role in numerous technological applications and show a great variety of magnetic properties. We have systematically investigated the structural properties, magnetic ground states, and fundamental electronic properties of 100 binary *d*-metal oxides using hybrid density functional methods and localized basis sets composed of Gaussian-type functions. The calculated properties are compared with experimental information in all cases where experimental data are available. The used PBE0 hybrid density functional method describes the structural properties of the studied *d*-metal oxides well, except in the case of molecular oxides with weak intermolecular forces between the molecular units. Empirical D3 dispersion correction does not improve the structural description of the molecular oxides. We provide a database of optimized geometries and magnetic ground states to facilitate future studies on the more complex properties of the binary *d*-metal oxides.

## 1. Introduction

*d*-metal oxides play a crucial role in many technological applications [1,2,3,4,5,6,7,8]. In particular, they find use in electronics [2,3,5], thermoelectrics [6,8], and applications related to their magnetic properties [7]. In addition to bulk metal oxide materials, oxide thin films possess unique properties due to their thickness-dependent properties which are widely known in catalysis [1,2,3,4]. Many of the *d*-metal oxides are magnetic, which complicates both experimental and computational studies. For example, magnetic structures of the *d*-metal oxides cannot be solved by ordinary X-ray techniques but require neutron diffraction or special techniques such as resonant X-ray scattering. In computational studies, open-shell magnetic compounds pose a challenge for methods based on density functional theory (DFT).

It is well known that DFT methods such as DFT-PBE, where the exchange-correlation functional is based on the generalized gradient approximation (GGA), fail in describing magnetic and electronic structures of strongly correlated *d*-metal oxides, sometimes even leading to a wrong magnetic ground state [9,10,11,12,13,14,15,16]. Even in the case of diamagnetic *d*-metal oxides such as Cu_2_O, DFT-GGA may describe the electronic properties rather poorly, resulting in a poor description of other properties such as phonons [17]. These challenges arise from the self-interaction error of DFT-GGA, resulting from the over-delocalization of the electrons in the metal *d* orbitals, in particular 3*d* orbitals [9,18,19,20,21]. As a result, the electronic structure can be even qualitatively wrong. This problem can be partially solved by using the Hubbard parameter (*U*) which localizes the electrons on the *d*-metal atoms [22]. However, even GGA + *U* underestimates band gaps of *d*-metal monoxides [18,23]. Furthermore, in addition to the problem with the *d*-metal orbitals, a similar issue with over-delocalization affects the oxygen 2*p* orbitals; in which case, the *U* correction does not help to overcome the problem [24]. Recently, promising results on magnetic La_2_CuO_4_ and VO_2_ were obtained by meta-GGA DFT-SCAN functional [25,26,27]. However, at the same time, it was shown that the treatment of the electronic structure of semiconducting and insulating materials by DFT-SCAN are typically not improved over DFT-GGA [28,29,30].

Hybrid density functionals that incorporate some exact exchange are known to significantly correct the self-interaction error, leading to correct description of the electronic and magnetic structures [15,18,20,21,24,31,32,33]. In particular, the main improvement of hybrid functionals over GGA functionals is the correct treatment of the valence bands near the Fermi level, leading to correct localization of the electrons [15,21]. The structural and electronic properties are typically described reasonably well, even if the band gap is generally overestimated [34,35]. Approaches to further improve the band gap prediction of hybrid functionals have also been suggested: dielectric-dependent hybrid functionals do not show real improvement, but the application of so-called charge transition level scheme leads to the further improvement of the predicted band gaps [36,37]. In principle, it is possible to tune the band gap predictions by tuning the amount of exact exchange, but such an empirical approach deteriorates the predictive power of the methodology [21]. In general, hybrid functionals with about 25% exact exchange such as PBE0 have been shown to describe *d*-metal oxides and their magnetic structures reasonably well [15,21,24,31]. The screened hybrid functionals such as HSE06 are another very commonly used approach in solid-state DFT studies, and they have been shown to predict band gaps of *d*-metal oxides and dichalcogenides with good accuracy [38].

Even though a vast number of computational studies on binary *d*-metal oxides have been reported in the literature, most of them have included only some subset of the binary *d*-metal oxides, and a comparison of the results is complicated by the variety of used computational methodologies. A comprehensive dataset of the structural properties and magnetic ground states of binary *d*-metal oxides, obtained with a DFT method that can properly describe the electronic structures of strongly correlated oxides, would facilitate future studies on more complex properties and eventual material applications. As an example of data analytics enabled by such datasets, Posysaev et al. recently investigated the oxidation states of a number of binary oxides taken from the AFLOW library [39]. Examples of physical and transport properties that can be nowadays accessed routinely with hybrid DFT methods are elastic, dielectric, piezoelectric, and thermoelectric properties [40].

Here, we present a comprehensive computational investigation of *d*-metal oxides known at the atmospheric pressure by using the hybrid DFT-PBE0 method (see Materials and Methods for computational details). We focus on binary *d*-metal oxides such as Fe_2_O_3_ and CuO and rule out ternary *d*-metal oxides such as FeTiO_3_ or CoTiO_3_. We studied in total 100 binary *d*-metal oxides, reporting their structural properties and magnetic ground states, including magnetic ground states at 0 K for materials that were reported to be paramagnetic at room temperature. We also investigate the effect of DFT-D3 dispersion correction on structural properties of molecular *d*-metal oxides [41]. We report the performance of the DFT-PBE0 method for binary *d*-metal oxides and provide a freely available dataset that enables further studies on their spectroscopic, mechanical, dielectric, and transport properties.

## 2. Results and Discussion

### 2.1. General Overview of the Results

We considered in total 100 binary d-metal oxides that are known to exist at the atmospheric pressure. We distinguished the studied oxides by their structural formula and Pearson symbol. Several *d*-metal oxides have high-temperature polymorphs which were included if they possessed an ordered crystal structure. High-pressure modifications were excluded from the present study. In the main text, we discuss only the magnetic oxides in detail, while results for nonmagnetic oxides are provided in the Supplementary Materials.

Table 1 lists the Pearson symbols, space groups, magnetic ground states, magnetic moments, and band gaps of the studied magnetic *d*-metal oxides. As discussed above, it is known that hybrid DFT methods may typically overestimate band gaps. Furthermore, our calculated band gaps are for perfect single crystals at 0 K, while the experimental band gaps are typically reported for room temperature and might depend significantly on the sample type (single crystal, polycrystalline, and thin film). Therefore, qualitative comparisons with the experimental results (insulating vs. metallic nature) are here more relevant than quantitative comparisons.

**Figure 1:**
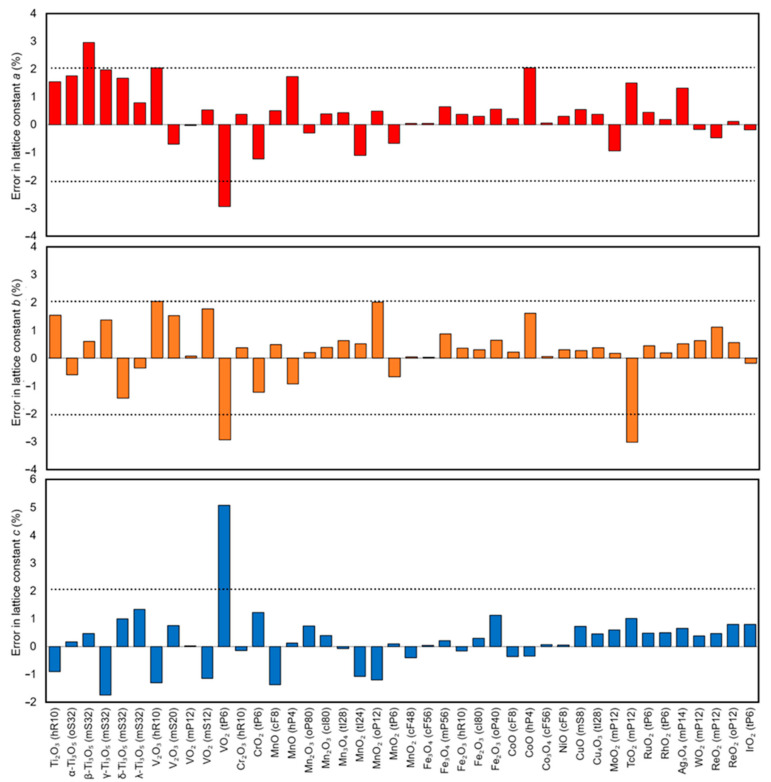
Errors in the optimized DFT-PBE0/TZVP lattice constants in comparison with the experimental lattice constants of the studied magnetic *d*-metal oxides. ReO_2_ (*tP*6) is not included in the plot (see text for details).

The overall performance of the DFT-PBE0/TZVP level of theory in reproducing lattice constants of the magnetic binary *d*-metal oxides is illustrated in Figure 1. In the lattice parameter comparisons for magnetic structures, the nonmagnetic experimental unit cell has been transformed so that it corresponds to the calculated magnetic unit cell. All reported magnetic moments are spin-only values without any orbital contributions, which can lead to some deviations from experimentally determined magnetic moments.

Generally, the DFT-PBE0/TZVP level of theory describes the structures of the studied magnetic *d*-metal oxides with good accuracy: mean absolute error (MAE) of the optimized lattice constants is 0.8%, and mean error (ME) is 0.3%. For comparison, using a smaller SVP basis set results in MAE of 0.9% and ME of 0.1%. The smaller SVP basis set thus appears to benefit from some cancellation of errors. For the whole set of the structures, including nonmagnetic *d*-metal oxides, MAE is 1.1% for TZVP basis set and 1.0% SVP basis sets, while ME is 0.6% for TZVP and 0.3% for SVP. The following metal oxides with abnormally large errors of more than 10% in the lattice constants are omitted in these statistics as outliers and discussed in the text: ReO_2_ (*tP*6), HgO_2_ (*mS*6), and HgO_2_ (*oP*12).

Out of the magnetic metal oxides investigated here, 16 are described in the literature as antiferromagnets. We were able to find an antiferromagnetic ground state for all of them. The antiferromagnetic configuration was described in the literature in full detail for 12 out of the 16 AFM oxides, and our results reproduced all these reported configurations, except for the helical configuration of MnO_2_ (*tP*6) or β-MnO_2_. Of the four systems that are ferrimagnetic according to the literature, three had the ferrimagnetic lowest-energy configurations, but for one (Fe_2_O_3_ *oP*40 or ε-Fe_2_O_3_), we found an antiferromagnetic ground state. Our ground state gives the same magnetic configuration as reported for the ferrimagnet, but the magnetic moments do not have values matching the ferrimagnetic configuration. The one experimentally ferromagnetic oxide (CrO_2_) was also reproduced. Of the 13 paramagnetic systems, our results predict an antiferromagnetic 0 K configuration for 10 and ferromagnetic for three oxides.

Concerning the predicted band gaps, DFT-PBE0 shows behavior that has been previously discussed in detail in the literature [33]. For systems where the experimental band gap is smaller than 1 eV, DFT-PBE0 typically significantly overestimates the band gap. This is evident especially in the case of titanium and vanadium oxides studied here. For band gaps between 2 and 5 eV, DFT-PBE0 produces more reasonable estimates. The comparisons of experimental and calculated band gaps are complicated by the fact that material defects such as vacancies can affect the band gap of the oxides significantly. Metal oxides often show, for example, nonstoichiometry, and some oxides such as TiO were even excluded from the study due to their significant nonstoichiometry. Finally, the band gaps discussed here were obtained simply as the fundamental 0 K energy gap between the highest occupied and lowest unoccupied bands, and both excitonic and finite temperature effects were neglected. It would, in principle, be possible to improve the agreement with experiments by tuning what is among the exact exchange for each material, but we avoided any empirical parametrization to obtain an overview of the performance of nonempirical PBE0 across the whole d-block.

We first discuss the results for the binary 3*d* metal oxides, followed by 4*d* and 5*d* oxides. Within each period, the *d*-metal oxides are discussed in order from group 3 to group 12. Additionally, we separately discuss molecular *d*-metal oxides and several mercury oxides.

Some binary *d*-metal oxides were excluded based on the following reasons: La_2_O_3_ (Pearson symbol *hP*5) is a high-T phase stable at > 2303 K and has occupancy of 0.5 at all sites; La_2_O_3_ (*cI*5) has an occupancy of 0.5 for oxygen atoms; TiO (*mS*20) and TiO (*cF*8) are nonstoichiometric; VO (*cF*8) is nonstoichiometric; ε-MnO_2_ (*hP*3) has 0.5 occupancy on Mn sites; in the case of Fe_3_O_4_ (*mS*224), the reduced structure Fe_3_O_4_ (*mP*56) was calculated instead; γ-Fe_2_O_3_ (*cP*56) has occupancy of 0.35 at a Fe site; MoO_3_ (*mP*16) has 0.5 occupancy on several sites; and TaO_2_ (*tP*6) is nonstoichiometric.

### 2.2. Magnetic Binary 3D-Metal Oxides

**Figure 2:**
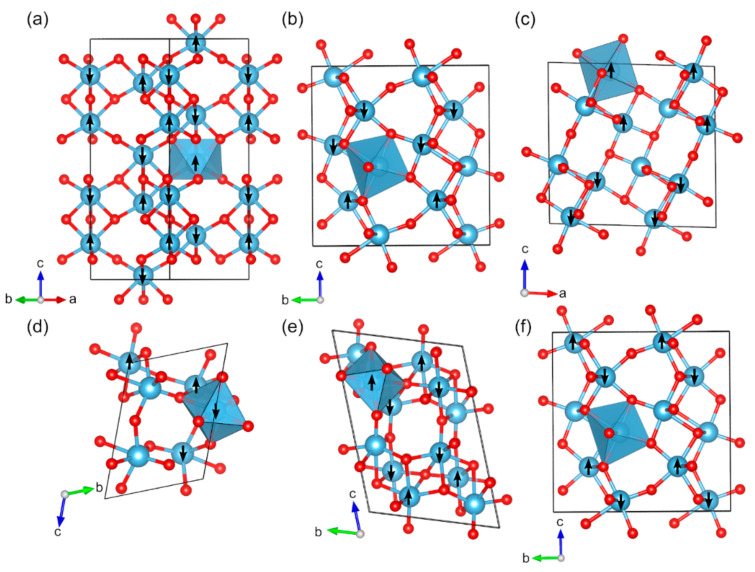
Optimized structures of studied magnetic binary titanium oxides (red: O, blue: Ti): (**a**) Ti_2_O_3_ (*hR*10), (**b**) α-Ti_3_O_5_ (*oS*32), (**c**) β-Ti_3_O_5_ (*mS*32), (**d**) γ-Ti_3_O_5_ (*mS*32), (**e**) δ-Ti_3_O_5_ (*mS*32), and (**f**) λ-Ti_3_O_5_ (*mS*32). The directions of the magnetic moments are illustrated by black arrows. Coordination octahedra of Ti atoms are shown in blue color.

Six of the studied titanium oxides are magnetic (Figure 2). Ti(III) oxide, Ti_2_O_3_ (*hR*10), adopts the trigonal corundum structure with space group *R*-3*c* (no. 167) [89]. Taking the magnetic structure into account changes the space group to subgroup *R*3*c* (161) (Figure 2a). There are no experimental data on the magnetic nature of Ti_2_O_3_. We found the AFM configuration to be the ground state of Ti_2_O_3_ with magnetic moments of 0.9 µ_B_, whereas previous calculations by the screened exchange hybrid DFT described Ti_2_O_3_ as a diamagnetic structure [90]. In the same paper, it was mentioned that the ground state of the Ti_2_O_3_ is experimentally determined to be diamagnetic at a low temperature. The lattice parameters of the calculated Ti_2_O_3_ (*hR*10) match the experimental values well with a difference of +1.6% for *a* and –0.9% for *c*. The calculated band gap of 2.7 eV is clearly overestimated compared to the experimental value of 0.1 eV [42]. However, harmonic frequency calculations performed on the structure revealed imaginary frequencies. Scanning along the imaginary modes reduced the symmetry of the antiferromagnetic structure to space group *C*1*c*1 (no. 9). This structure was also observed to be energetically more favorable than the higher symmetry one (by about 2 kJ/mol per atom), with a band gap of 2.5 eV and magnetic moment of 1.0 µ_B_.

Ti(III/IV) oxide, α-Ti_3_O_5_, (*oS*32) crystallizes in an orthorhombic crystal structure with space group *Cmcm* (no. 63) (Figure 2b) [91]. Taking the magnetic ordering into account changes the space group to the subgroup *Cm* (8). α-Ti_3_O_5_ exists at temperatures higher than 460 K, while below 460 K it transforms to β-Ti_3_O_5_. There is little experimental data available on the magnetic and electronic properties of α-Ti_3_O_5_. The lattice parameters are described with good accuracy compared to the experiment: the calculated lattice constants differ from the experimental values by +1.8% for *a*, −0.6% for *b*, and +0.2% for *c*. We identified ferrimagnetic spin-ordering for α-Ti_3_O_5_ with magnetic moments of 1.0 µ_B_ for some Ti^3+^ atoms and nonmagnetic Ti^4+^ (Table 1). The calculated band gaps (2.0 eV) cannot be compared with the literature as there are no previous experimental or computational data, but the material has been reported to be a semiconductor [92].

β-Ti_3_O_5_ (*mS*32) adopts monoclinic crystal structure with space group *C*2/*m* (no. 12) [93]. For the magnetic ordering, the space group is changed to the subgroup *Cm* (8) (Figure 2c). We found an antiferromagnetic configuration with magnetic moments of 0.9 and 1.0 µ_B_ on Ti^3+^ atoms to be the most favorable energetically, whereas no experimental data are available on the magnetic ordering. Based on our calculations, β-Ti_3_O_5_ (*mS*32) is an insulator with a band gap of 1.3 eV, compared to experimentally measured 0.14 eV [43]. In comparison to the experiment, the optimized lattice constants of the β-Ti_3_O_5_ (*mS*32) show differences of +3.0% for *a*, +0.6% for *b*, and +0.5% for *c*.

Another Ti(III/IV) oxide, γ-Ti_3_O_5_ (*mS*32), has a monoclinic crystal structure with space group *I*2/*c* (15) (Figure 2d) [44]. γ-Ti_3_O_5_ is formed from β-Ti_3_O_5_ at ~250 K and further transforms to δ-Ti_3_O_5_ below 237 K [94]. Based on our calculations, γ-Ti_3_O_5_ is an insulator with band gap of 2.3 eV, whereas it has been reported to be metallic based on the experimental data [44]. By studying different magnetic configurations, we found that antiferromagnetic γ-Ti_3_O_5_ (*mS*32) is the most favorable configuration, energetically speaking. The magnetic ground state has four Ti^3+^ atoms with localized spins and two nonmagnetic Ti^4+^ atoms (Table 1). In comparison to the experiment, the optimized lattice constants of γ-Ti_3_O_5_ (*mS*32) show differences of +2.0%, +1.4%, and −1.7% for *a*, *b*, and c, respectively.

δ-Ti_3_O_5_ (*mS*32) adopts monoclinic crystal structure with space group *P*2/*a* (no. 13) [44]. The space group of the magnetically ordered structure is *P*-1 (no. 2) (Figure 2e). Based on our calculations, δ-Ti_3_O_5_ (*mS*32) is an insulator with a band gap of 2.4 eV, and clearly overestimated compared to 0.07 eV from experimental studies [44]. The lattice parameters compare well with experimentally known crystal structure, with differences of 1.7% for *a*, −1.4% for *b*, and 1.0% for *c.* As for other Ti_3_O_5_ phases, there are no experimental data on magnetic moments of δ-Ti_3_O_5_ (*mS*32), but it is estimated to be 1.0 µ_B_ for Ti^3+^ atoms.

Recently, synthesis of a new structure, λ-Ti_3_O_5_ (*mS*32) was reported that crystallizes in monoclinic crystal structure with space group *C*2/*m* (no. 12) [43]. We found antiferromagnetic configuration to be the energetically most favorable for λ-Ti_3_O_5_ (*mS*32), arising from magnetically ordered structure in subgroup *Cm* (no. 8) (Figure 2f). Calculated magnetic moments are 1.0 µ_B_ for Ti^3+^ atoms. Even though experimentally the material was reported to be metallic, our calculations showed band gap of 1.7 eV. Such discrepancy may be due to the experimental conditions: a photoreversible metal-semiconductor phase transition. Also, nanoparticles (ca. 25 nm.) were studied and the results can be different compared to single-crystalline bulk material. The optimized lattice constants of λ-Ti_3_O_5_ (*mS*32) differ from experimental data by +0.8% for *a*, −0.3% for *b*, and 1.3% for *c.*

**Figure 3:**
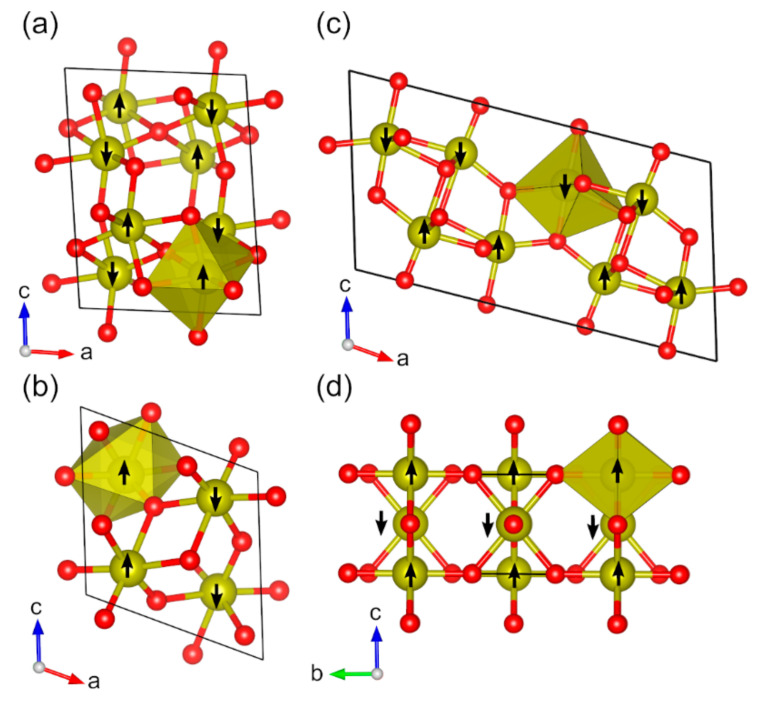
Optimized structures of studied magnetic binary vanadium oxides (red: O, yellow: V): (**a**) V_2_O_3_ (*mS*20), (**b**) VO_2_ (*mP*12), (**c**) VO_2_ (*mS*12), and (**d**) VO_2_ (*tP*6). The directions of magnetic moments are illustrated by black arrows. Coordination octahedra of V atoms are shown in yellow color.

For vanadium, we investigated five magnetic binary oxides (Figure 3). Two V(III) oxides are known: V_2_O_3_ (*hR*10) and V_2_O_3_ (*mS*20). V_2_O_3_ (*hR*10) is stable above 155 K and V_2_O_3_ (*mS*20) below 155 K [95]. The V_2_O_3_ (*hR*10) modification crystallizes in the trigonal corundum structure with space group *R*-3*c* (no. 167) and has been described in detail in a recent DFT study [31,69,95,96,97]. V_2_O_3_ (*mS*20) has a monoclinic crystal structure with space group *I*2/*a* (no. 15) [45,98]. In line with experimental data, the magnetic ground state of V_2_O_3_ (*mS*20) was found to be AFM configuration with a calculated spin magnetic moment of 2.0 µ_B_ (exp. value 1.2 µ_B_) (Table 1). The AFM ground state can be realized in the subgroup *P*2*c* (no. 13) (Figure 3a). Optimized lattice parameters match the experimental values with good accuracy, lattice parameters differing by −0.7%, +1.5%, and +0.8% for *a*, *b*, and *c*, respectively. The calculated band gap is 2.8 eV, while a clearly smaller gap of 0.6 eV has been reported experimentally [46].

Three different VO_2_ modifications are known: VO_2_ (*mP*12)*,* VO_2_ (*mS*12), and VO_2_ (*tP*6) [99,100]. VO_2_ (*mP*12) crystallizes in the space group *P*2_1_/*c* (no. 14), the symmetry being lowered to subgroup *P*2_1_ (no. 4) for the magnetically ordered structure (Figure 3b). VO_2_ (*mS*12) has a monoclinic crystal structure with space group *C*2/*m* (no. 12), while taking the magnetic ordering into account lowers the symmetry to subgroup *Cm* (no. 8) (Figure 3c). The tetragonal VO_2_ (*tP*6) modification crystallizes in the space group *P*4_2_/*mnm* (no. 136), and subgroup *Cmmm* (no. 65) was used to describe the magnetic ordering (Figure 3d).

Overall, all three VO_2_ modifications have been experimentally characterized to be paramagnetic [101]. VO_2_ (*mP*12) is stable below 340 K, while VO_2_ (*tP*6) is a high*-*temperature modification that is stable above 340 K. Experimentally, the high-temperature VO_2_ (*tP*6) modification was found to be metallic, whereas our 0 K calculations show a 2.8 eV band gap [99]. In line with the experimental data, we found VO_2_ (*mP12*) to be lower in energy compared to VO_2_ (*tP*6) at 0 K (by 1.4 kJ mol^−1^ per atom) [102]. The calculated band gap of VO_2_ (*mP*12) is 3.0 eV, whereas experimentally it is estimated to be ca. 0.6–0.7 eV (Table 1). VO_2_ (*mS*12) is known to be stable at high pressure and at a zero-strain triple point at 338 K [103]. The lattice constants differ from experimental values by 0.0%, +0.1%, and 0.4% for VO_2_ (*mP*12) and by +0.5%, +1.8%, and −1.1% for VO_2_ (*mS*12). VO_2_ (*tP*6) modification shows relatively large differences of −2.9% for *a* and b and 5.1% for *c* compared to experimental data. This is likely due to the fact the VO_2_ (*tP*6) is a high-temperature modification. Magnetic moments of VO_2_ (*mP*12) were reported in a computational study to be −1 µ_B_, which is in line with our calculated value of 1.1 µ_B_ (Table 1) [104].

In the case of chromium, we investigated two magnetic binary oxides. Cr(III) oxide, Cr_2_O_3_ (*hR*10), crystallizes in the corundum structure type with space group *R*-3*c* (no. 167) [48]. Cr_2_O_3_ (*hR*10) adopts an AFM spin configuration below the Neel temperature of 309 K, and the magnetically ordered structure in subgroup *R*3*c* (no. 161) is identical to Ti_2_O_3_ (*hR*10) shown in Figure 2a. The lattice constants of the optimized structure match the experimental data well, with a difference of less than 0.5%. The calculated band gap is 5.1 eV, which is larger in comparison with 3.2–3.4 eV from experimental measurements (Table 1). The magnetic moments of the AFM structure are in good agreement with experimental data (3.0 µ_B_ calc. and 2.7 µ_B_ exp.).

**Figure 4:**
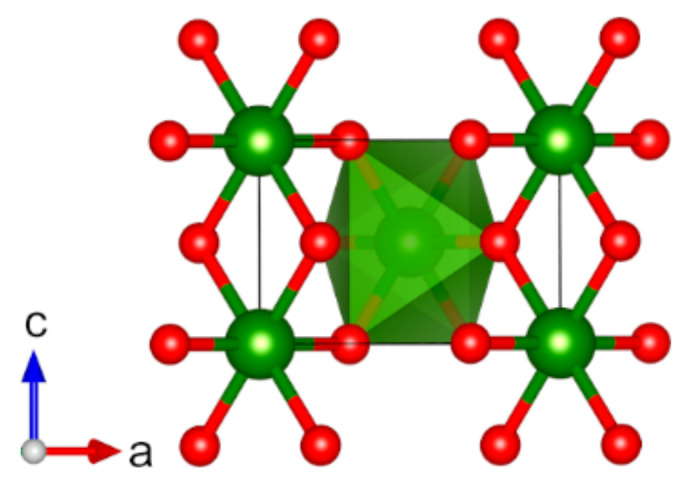
Optimized structure of CrO_2_ (*tP6*) (red: O, green: Cr). Spins are aligned along *c* axis and not visualized. Coordination octahedron of Cr is shown in green color.

Cr(IV) oxide, CrO_2_ (*tP*6), crystallizes in the rutile structure type with space group *P*4_2_/*mnm* (no. 136) [105]. The magnetic structure is known to be ferromagnetic with a Curie temperature of 386.5 K (Figure 4), and the material is known to be a metallic conductor [105,106,107,108]. The calculated magnetic moment is in good agreement with the experimental value (2.4 µ_B_ calc. vs. 2.01 µ_B_ exp.) (Table 1), and the lattice parameters of the optimized structure are in line with experimental data (*a* and *c* differ by +1.2%).

**Figure 5:**
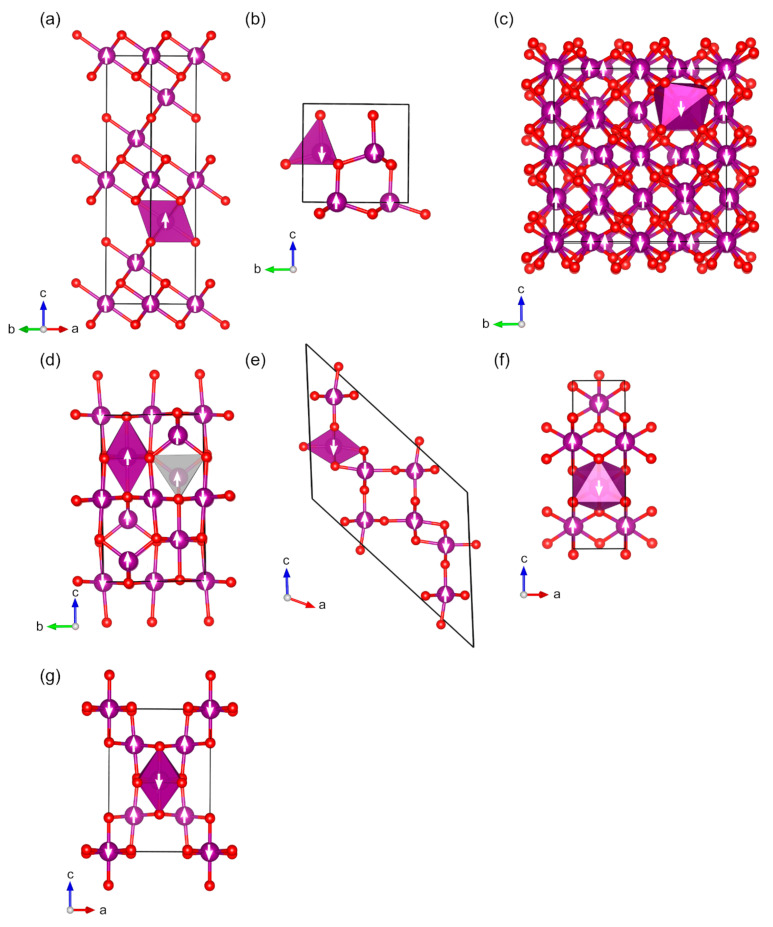
Optimized structures of studied magnetic binary manganese oxides red, O; violet: Mn: (**a**) MnO (*cF*8), (**b**) MnO (hP4), (**c**) Mn_2_O_3_ (*oP*80), (**d**) Mn_3_O_4_ (*tI*28), (**e**) MnO_2_ (*tI*24), (**f**) MnO_2_ (*oP*12), and (**g**) MnO_2_ (*cF*48). The directions of magnetic moments are illustrated by white arrows. Coordination octahedra and tetrahedra of Mn atoms are shown in violet and light grey colors, respectively.

For manganese, we studied nine magnetic binary oxides (Figure 5). Mn(II) oxide, MnO (*cF*8), crystallizes in the rock salt structure type with space group *Fm*-3*m* (225). For the magnetically ordered structure, the symmetry is reduced to subgroup *R*-3*m* (no. 166) (Figure 5a) [74]. The magnetic ground state of MnO is known to be AFM with a Néel temperature of about 122 K [109]. The lattice parameters of the calculated MnO (*cF*8) structure are in good agreement with experimental data: the difference is +0.5% and –1.4% for *a* and *c*, respectively. The calculated magnetic moment, 4.8 µ_B_, is in line with the experimental value of 4.58 µ_B_ (Table 1), and the calculated band gap is also in the range of experimentally measured band gaps (3.9 eV calc. and 3.6–4.2 eV exp.). Hexagonal polymorph of MnO crystallizes in the wurtzite structure type with space group *P*6_3_*mc* (186). The magnetic ground state has not been experimentally determined, but previous computational studies report an antiferromagnetic structure [110,111]. The space group symmetry of our calculated antiferromagnetically ordered structure is reduced to subgroup *Pmc*2_1_ (no. 26). The calculated band gap of 3.0 eV is smaller than that of the cubic polymorph, while the magnetic moment is the same at 4.8 µ_B_. Lattice parameter differences are +1.7% for *a*, –0.9% % for *b*, and +0.1% for *c*.

Two magnetic Mn(III) oxides are known: Mn_2_O_3_ (*oP*80) and Mn_2_O_3_ (*cI*80) [112,113]. The space groups of Mn_2_O_3_ (*oP*80) and Mn_2_O_3_ (*cI*80) are *Pbca* (no. 61) and *Ia*-3 (no. 206), respectively. Orthorhombic Mn_2_O_3_ (*oP*80) is stable below 302 K, and above this temperature, the cubic Mn_2_O_3_ (*cI*80) modification becomes more stable. Only Mn_2_O_3_ (*oP*80) is shown in Figure 5c as the structures look very similar and only differ by the magnetic ordering. Mn_2_O_3_ (*oP*80) is experimentally known to be antiferromagnetic, whereas Mn_2_O_3_ (*cI*80) is considered to be paramagnetic [55,56,114]. Based on our calculations, the Mn_2_O_3_ (*cI*80) prefers a ferromagnetic spin configuration at 0 K (Table 1). The lattice parameters are described with good accuracy: the difference between the optimized and experimental lattice constants is less than 0.7%. A direct comparison of the electronic structure of the Mn_2_O_3_ modifications with experiments is not possible due to the absence of experimental data on bulk materials. Band gaps of Mn_2_O_3_ (*oP*80) were estimated to be 2.17 and 2.4 eV for nanoparticles and thin films, respectively, whereas our calculated band gap is 3.0 eV [57,58]. Based on our calculations, Mn_2_O_3_ (*cI*80) is a metallic conductor, whereas some experimental studies of nanostructured modifications suggest that the material possesses a band gap (1.24 or 1.8 eV) [57,115]. In this case, however, it is difficult to compare the results as the experimental studies also found that the band gap of Mn_2_O_3_ (*cI*80) is directly correlated with the size of the nanoparticles (increased size leads to a smaller band gap). Experimentally measured magnetic moments are only available for Mn_2_O_3_ (*oP*80), and they have been reported as 2.3–3.9 µ_B_ (calculated values are 3.9 and 4.0 µ_B_).

Mn(II/III) oxide, Mn_3_O_4_ (*tI28*), has a tetragonal crystal structure with space group *I*4_1_/*amd* (no. 141) (Table 1) [116]. Mn_3_O_4_ is known to adopt a ferrimagnetic spin configuration at the room temperature, with the magnetically ordered structure having space group *Imma* (no. 74) (Figure 5d) [117]. The lattice parameters of the optimized structure are larger than the experimental values only by +0.4%, +0.6%, and −0.1% for *a*, *b*, and *c*, respectively. There is no experimental information available on the magnetic moments, but our results are in good agreement with a previously reported computational studies [118,119]. The band gap of Mn_3_O_4_ nanoparticles was found to be in range of 1.77–2.72 eV depending on the size, whereas our calculated bulk band gap is 3.2 eV [59].

For Mn(IV), we studied four polymorphs: MnO_2_ (*tI*24), MnO_2_ (*oP*12), MnO_2_ (*tP*6), and MnO_2_ (*cF*48) [120,121,122,123]. MnO_2_ (*tI*24), also known as α-MnO_2_, crystallizes in space group *I*4*/m* (no. 87), and for the magnetically ordered structure, the symmetry is lowered to space group *C*2/*m* (no. 12) (Figure 5e). MnO_2_ (*oP*12) polymorph (γ-/R-MnO_2_) adopts an orthorhombic structure with space group *Pnam* (no. 62), with a magnetically ordered structure in space group *Pmc*2_1_ (no. 26) (Figure 5f). MnO_2_ (*tP*6) (β-MnO_2_) crystallizes in the rutile structure type, space group *P*4_2_/*mnm* (136), and the magnetic structure in the space group *Cmmm* (no. 65) (identical to VO_2_ (*tP*6), Figure 3d). MnO_2_ (*cF*48) (λ-MnO_2_) crystallizes in the cubic space group *Fd*-3*m* (227), whereas the magnetic structure is orthorhombic with space group *Imma* (no. 74) (Figure 5g). The lattice parameters of the optimized structures match the experimental data very well: the typical difference between calculated and experimental lattice constants is less than 1%. MnO_2_ (*tP*6) possesses a helical magnetic configuration below 92 K [56,124,125], whereas all other polymorphs are antiferromagnetic (the Néel temperatures of MnO_2_ (*tI*24) and MnO_2_ (*cF*48) are 24.5 and 32 K, respectively) [63,126,127]. The only available experimentally measured magnetic moments are for MnO_2_ (*cF*48): 3.1 µ_B_ calc. compared to 2.78 µ_B_ exp. (Table 1). The data on band gaps of bulk structures are also very limited, and we only found a gap of 0.3 eV reported for MnO_2_ (*tP*6) (2.1 eV calc.). The band gap of thin films of MnO_2_ (*cF*48) was estimated from the experiment to be in the range of 1.7–3.5 eV, while the calculated band gap for the bulk structure is 3.7 eV [64]. The band gap of nanoflakes of MnO_2_ (*oP*12) was estimated to be 2.57 eV, whereas the calculated bulk band gap is 3.5 eV [61].

**Figure 6:**
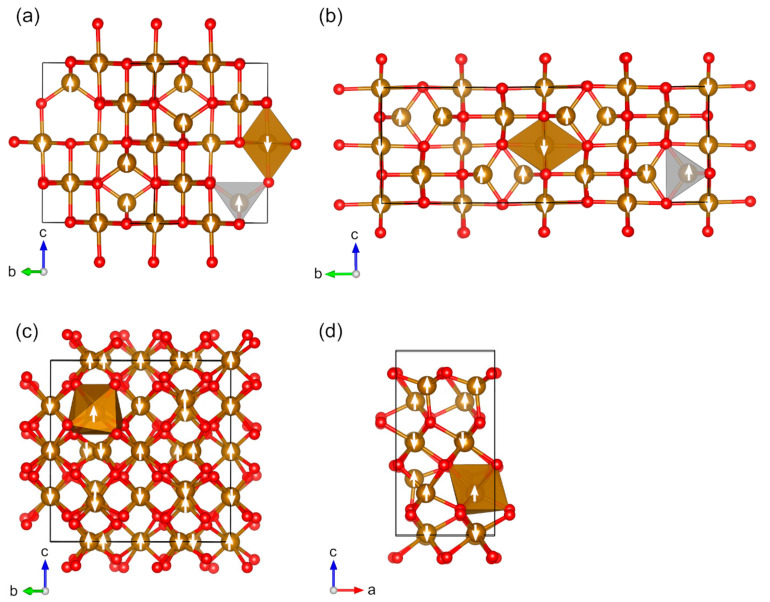
Optimized structures of studied magnetic binary iron oxides (red: O, brown: Fe): (**a**) Fe_3_O_4_ (*cF56*), (**b**) Fe_3_O_4_ (*mP56*), (**c**) Fe_2_O_3_ (*cI80*), and (**d**) Fe_2_O_3_ (*oP40*). The directions of magnetic moments are illustrated by white arrows. Coordination octahedra and tetrahedra of Fe atoms are shown in brown and light-grey colors, respectively.

For iron, we investigated five magnetic binary oxides. Two Fe(II/III) oxides are known: Fe_3_O_4_ (*cF*56) and Fe_3_O_4_ (*mP*56) [66,128]. Fe_3_O_4_ (*cF*56) crystallizes in space group *Fd*-3*m* (no. 227) (Figure 6a) and Fe_3_O_4_ (*mP*56) in space group *P*2/*c* (no. 13) (Figure 6b). Fe_3_O_4_ (*mP*56), which is stable below 125 K, is involved in the Verwey transition below 125 K from the cubic structure [129]. The lattice constants of the optimized Fe_3_O_4_ (*cF*56) structure exactly reproduce experimental values, whereas the Fe_3_O_4_ (*mP*56) shows differences of +0.6%, +0.9%, and +0.2% for *a*, *b*, and *c*, respectively. Fe_3_O_4_ (*cF*56) is known to be ferrimagnetic at the room temperature, and it is a metallic conductor [11,128,130]. Fe_3_O_4_ (*mP*56) is also a ferrimagnet [66]. Calculated atomic magnetic moments (4.0/4.2 µ_B_ for *cF*56 and 3.7–4.3 µ_B_ for *mP*56) are in good agreement with the experimental values (3.82 µ_B_ for *cF*56 and 4.17 and 4.44 µ_B_ exp. for *mP56*). The calculated band gap of Fe_3_O_4_ (*mP*56) is overestimated by being 1.6 eV in comparison to the experimentally determined 0.1 eV (Table 1).

We investigated three Fe(III) oxides: Fe_2_O_3_ (*hR*10), Fe_2_O_3_ (*cI*80), and Fe_2_O_3_ (*oP*40) [68,131,132]. The calculated lattice constants are in the good agreement with experimental values, with the largest difference being 0.9%. Fe_2_O_3_ (*hR*10), hematite or α-Fe_2_O_3_, is known to be antiferromagnetic with the Neel temperature of 955 K, and it has been described in detail in a recent computational study [31]. It crystallizes in the space group *R*-3*c* (no. 167), whereas the symmetry of the magnetically ordered structure is lowered to space group *R*-3 (no. 148). Fe_2_O_3_ (*cI*80), β-Fe_2_O_3_, is also known to be antiferromagnetic [133,134]. The space group of the AFM ground state is *Ia*-3 (no. 206), and the Neel temperature is 119 K (Figure 6c). To our knowledge, there are no experimental data available on the magnetic moments of Fe_2_O_3_ (*cI*80). The calculated band gap is overestimated to be 3.3 eV compared to the experimental value of 2.2 eV (Table 1). Fe_2_O_3_ (*oP*40), ε-Fe_2_O_3_, is ferrimagnetic with a Curie temperature of 495 K [135]. However, we found the antiferromagnetic configuration to be the energetically most favorable configuration (ferromagnetic configurations were 2.6–4.7 kJ mol^–1^ per atom higher in energy, see Supplementary Materials). Spins are correctly aligned in the structure, but Fe atoms in the tetrahedral sites have smaller magnetic moments than they would in a ferrimagnetic configuration. The space group of Fe_2_O_3_ (*oP*40) is *Pna*2_1_ (no. 33), and the magnetically ordered structure has the same space group (Figure 6d). The calculated band gap is estimated to be 4.2 eV, which is clearly overestimated in comparison to the experimental value being 1.6 eV. A comparison of estimated magnetic moments is not feasible as values are only available for nanoparticles.

**Figure 7:**
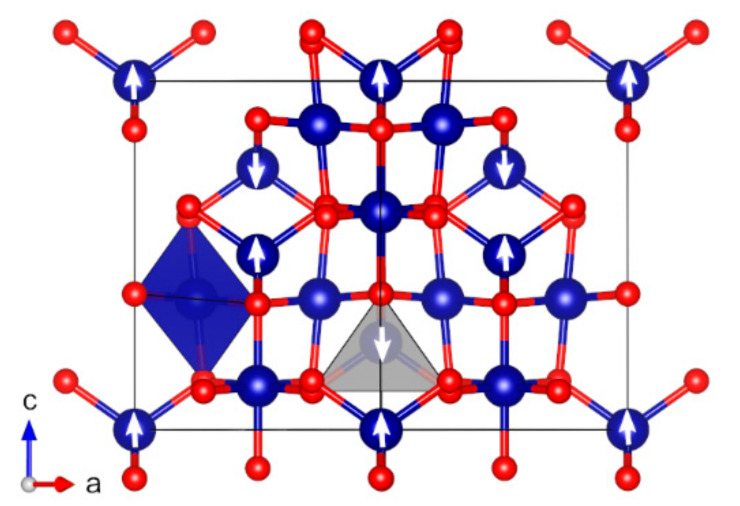
Optimized structure of Co_3_O_4_ (*cF*56) (red: O, dark blue: Co). The directions of magnetic moments are illustrated by white arrows. Coordination octahedra and tetrahedra of Co atoms are shown in dark-blue and light-grey colors, respectively.

Co(II) oxide, CoO (*cF*8), crystallizes in the rocksalt structure with space group *Fm*-3*m* (no. 225) [72,73,74]. Similar to MnO (*cF*8), the space group of the magnetically ordered structure is *R*-3*m* (no. 166). CoO (*cF*8) is described in detail in recent computational studies [31,136]. The hexagonal wurtzite polymorph of CoO, *P*63*mc* (no. 186), has an antiferromagnetic structure similar to hexagonal MnO [137], with the magnetical ordering lowering the space group to *Pmc*2_1_ (no. 26). The calculated band gap is 3.2 eV, which is smaller than the value of 4.5 eV of the cubic polymorph. The magnetic moment is 2.8 µ_B_. The lattice parameter differences compared to experiments are +2.0% for *a*, +1.6% for *b*, and −0.3% for *c*. Co(II/III) oxide. Co_3_O_4_ (*cF*56) has a cubic structure with space group *Fd*-3*m* (no. 227) [138]. The magnetic structure is antiferromagnetic with space group *F*-43*m* (no. 216) and shows a Neel temperature of 30 K (Figure 7) [75]. The lattice constants are almost identical to the experimental values with a difference of only 0.1%. The calculated magnetic moments of Co^2+^, 2.8 µ_B_, reproduce at least one reported experimental value of 3.0 µ_B_ (Table 1). The reports on Co_3_O_4_ band gap show a large variation from 0.74 even up to 4.4 eV, with the most recent studies suggesting a fundamental gap of about 0.8 eV [13,77]. Our calculated band gap of 4.0 eV is clearly overestimated in comparison to the values of less than the 1 eV suggested in recent studies. Singh et al. have shown that the electronic and magnetic properties of Co_3_O_4_ are very sensitive to the choice of the Hubbard parameter (for PBE + *U_eff_*) and the amount of exact exchange included in the HSE06 hybrid functional [13]. Other computational studies have also shown that the DFT+*U* calculations with *U* values calibrated to the experimental data are required to obtain a good agreement with experimental band gaps [139,140]. Co_3_O_4_ appears to be a very good benchmark case for any new nonempirical DFT methods.

Nickel(II) oxide, NiO (*cF*8), crystallizes in the rocksalt structure, and it has a similar antiferromagnetic structure as MnO (*cF*8) and CoO (*cF*8) [52,74,141,142]. NiO (*cF*8) is described in detail in recent computational studies [31,136].

**Figure 8:**
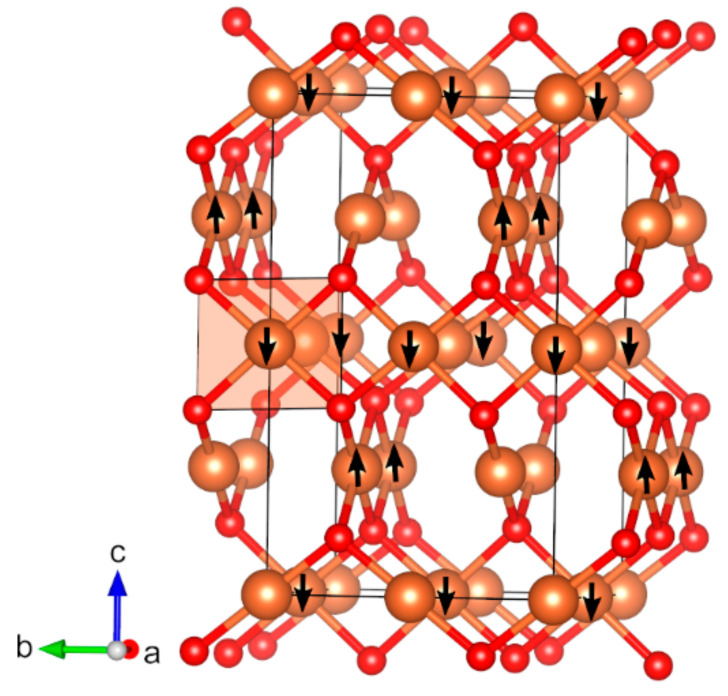
Optimized structure of Cu_4_O_3_ (*tI*28) (red: O, light brown: Cu). The directions of magnetic moments are illustrated by black arrows. Coordination square planar of Cu atoms that are shown is a light-brown color.

Copper(II) oxide, CuO (*mS*8), is known to have a monoclinic structure with space group *C*2/*c* (no. 15) and antiferromagnetic ground state [82,83,84,143,144]. The space group of the magnetic structure is *P*2_1_/*c* (no. 14), and the structure is described in detail in previous computational studies [31,35]. Copper(I/II) oxide, Cu_4_O_3_ (*tI*28), crystallizes in a tetragonal crystal structure with space group *I*4_1_/*amd* (no. 141), with the symmetry of the magnetic structure being reduced to the subgroup *Imma* (no. 74) (Figure 8) [145,146]. Cu_4_O_3_ (*tI*28) is known to be stable as an antiferromagnetic structure with the Neel temperature of 41 K [86]. Whereas the band gap of the optimized structure is overestimated compared with experimentally estimated (2.9 eV. calc. vs. −1.5 eV. exp.), the calculated magnetic moments of Cu^2+^ (0.7 µ_B_) are almost identical to experimental value of 0.66 µ_B_. The lattice parameters of the optimized structure also match the experimental data well, with differences of less than 0.5%.

### 2.3. Magnetic Binary 4D-Metal Oxides

Mo(IV) oxide, MoO_2_ (*mP*12), crystallizes in a monoclinic crystal structure with space group *P*2_1_/*c* (no. 14) [147]. It has been determined to be paramagnetic at room temperature [148,149]. We found an antiferromagnetic configuration to possess the lowest energy at 0 K for MoO_2_ (*mP*12) (identical to VO_2_ (*mP*12) and shown in Figure 3b). Therefore, the symmetry of the magnetic configuration is reduced to subgroup *P*2_1_ (no. 4). MoO_2_ (*mP*12) is a metallic conductor [148]. The lattice parameters of the optimized MoO_2_ (*mP*12) are in good agreement with the experimental data, with the differences < 1%. Estimated magnetic moments of Mo atoms are 1.1 µ_B_.

For radioactive Tc, a magnetic Tc(IV) oxide is known. TcO_2_ (*mP*12) is isostructural to MoO_2_ (*mP*12) with *P*2/*c* space group (no. 12) and subgroup *P*2_1_ (no. 4) for the magnetic configuration (identical to VO_2_ (*mP*12), Figure 3b) [150]. Overall, very few data on the TcO_2_ (*mP*12) are available. We found an antiferromagnetic ground state with magnetic moments of 2.7 µ_B_ on the metal atoms. Based on our results, TcO_2_ (*mP*12) possesses a band gap of 2.4 eV. Compared to the experimental crystal structure, the lattice parameters *a*, *b*, and *c* differ by +1.5%, −3.0%, and 1.0, respectively.

Ru(IV) oxide, RuO_2_ (*tP*6), is a rutile structure with space group *P*4_2_/*mnm* (no. 136) [151]. The symmetry of the magnetically ordered structure is lowered to subgroup *Cmmm* (no. 65) (identical to VO_2_ (*mP*12), Figure 3d). It was originally determined to be paramagnetic within 4–300 K [152], but based on recent experimental and computational work, it is an antiferromagnet with a Neel temperature over 300 K [88]. We found an antiferromagnetic ground state with magnetic moment of 1.5 µ_B_, whereas the experiments showed small magnetic moments of 0.05 µ_B_. The lattice parameters of the optimized structure are in good agreement with the experimental data (with differences less than 0.5%).

Rh(IV) oxide, RhO_2_ (*tP*6), also adopts the rutile structure with space group *P*4_2_/*mnm* (no. 136) [153]. We found a ferromagnetic ground state (identical to CrO_2_, Figure 4). Based on our calculations, RhO_2_ is metallic (Table 1) [154]. The experimental data on electronic and magnetic properties are limited, and it has only been mentioned that RhO_2_ (*tP*6) should be paramagnetic at room temperature [155]. The structural properties are in good agreement with the experimental data: the lattice parameters differ by +0.2% and +0.5% for *a* and *c*, respectively.

**Figure 9:**
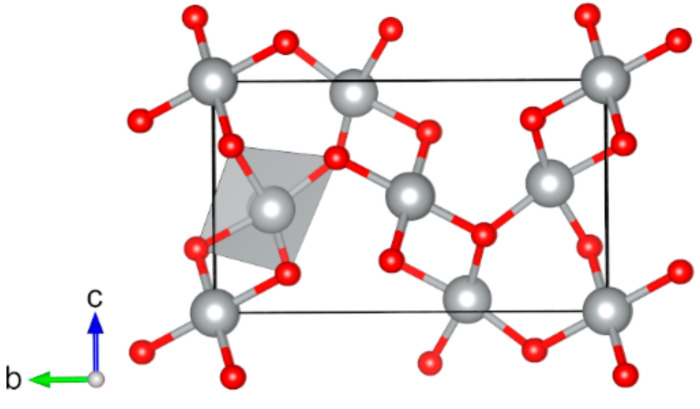
Optimized structure of Ag_3_O_4_ (*mP*14) (red: O, light grey: Ag). Spins are aligned up along *c* axis and not visualized. Square planar coordination of Ag atoms is shown in a light-grey color.

Ag(II/III) oxide, Ag_3_O_4_ (*mP*14), crystallizes in a monoclinic crystal structure with space group *P*2_1_/*c* (no. 14) (Figure 9) [156]. The lattice parameters match the experimental data well: the differences between the optimized and experimental lattice constants are +1.2%, +0.5%, and +0.6% for *a*, *b*, and *c*, respectively. Ag_3_O_4_ has been reported to be paramagnetic above 70 K [156,157]. At 0 K, we found the ferromagnetic ground state with Ag^3+^ magnetic moments of 0.2 µ_B_. We found Ag_3_O_4_ to be the metallic conductor (Table 1). The electronic structure of Ag_3_O_4_ has been described as magnetic in the literature [158], but no further details on how it was determined or other experimental data were provided.

### 2.4. Magnetic Binary 5D-Metal Oxides

W(IV) oxide, WO_2_ (*mP*12), has a monoclinic crystal structure with space group *P*2_1_/*c* (no. 14) [159]. The magnetically ordered structure has a lower symmetry with subgroup *P*2_1_ (no. 4) (identical to VO_2_ (*mP*12), Figure 3b). To our knowledge, there are no experimental or computational studies on the magnetic properties of WO_2_ (*mP*12). The paramagnetic ground state is mentioned in the book by Richards [155], which describes WO_2_ as a metallic compound. However, the experimental conditions of the measurements are not provided. Based on our calculations, the ground state of WO_2_ (*mP*12) is antiferromagnetic with magnetic moments of 0.4 µ_B_. The lattice parameters of the calculated structure are in good agreement with the experimental data, showing differences of −0.2% for *a*, +0.6% for *b*, and +0.4% for *c*.

**Figure 10:**
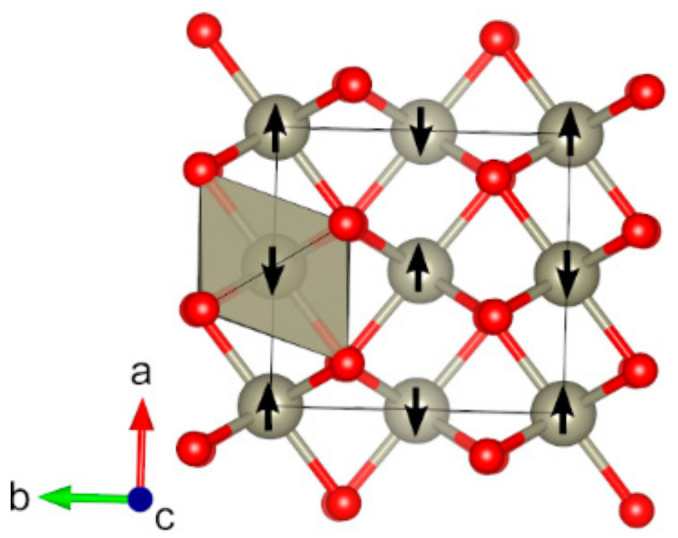
Optimized structure of ReO_2_ (*mP12*) (red: O, grey: Re). The directions of magnetic moments are illustrated by black arrows. Coordination octahedra of Re atoms is shown in grey color.

For rhenium, we investigated three Re(IV) magnetic oxides: ReO_2_ (*mP*12), ReO_2_ (*oP*12), and ReO_2_ (*tP*6). ReO_2_ (*mP*12) crystallizes in a monoclinic crystal structure with space group *P*2_1_/*c* (no. 14). For the magnetically ordered structure, the symmetry is lowered to subgroup *P*2_1_ (no. 4) (identical to VO_2_ (*mP*12) and shown in Figure 3b). Monoclinic ReO_2_ (*mP*12) structure is experimentally characterized to be paramagnetic below 573 K [160,161]. ReO_2_ (*oP*12) crystallizes in an orthorhombic crystal structure with space group *Pbcn* (no. 60) [162]. The symmetry of the magnetically ordered structure is lowered to space group *P*2_1_2_1_2 (no. 18) (Figure 10). It has been determined to be a metallic and paramagnetic compound between 4.2 K and the room temperature [148]. Based on our calculations at 0 K, ReO_2_ (*oP*12) has a band gap of 1.6 eV. We found an antiferromagnetic ground state with magnetic moments of 1.1 µ_B_. In agreement with our findings, a recent computational study showed that at 0 K, the structure adopts antiferromagnetic ordering [163]. ReO_2_ (*tP*6) adopts tetragonal crystal structure with space group *P*4_2_/*mnm* (no. 136) [162,164,165]. The symmetry of the magnetically ordered structure is lowered to subgroup *Cmmm* (no. 65) (identical to VO_2_ (*tP*6), Figure 3d). The lattice parameters of the optimized ReO_2_ (*mP*12) and ReO_2_ (*oP*12) structures are in good agreement with experimental data, showing differences of less than 1.1%. However, ReO_2_ (*tP*6) shows a difference of about 14% for the lattice constant *c*. Similar to tetragonal VO_2_, it is possible that tetragonal ReO_2_ (*tP*6) structure at 0 K is different from the experimental structure determined at a higher temperature (the material was synthesized at 693 K). Very limited information is available on tetragonal ReO_2_ (*tP*6); only one experimental/computational paper has been reported [166]. DFT-LDA + *U* calculations suggested that the ReO_2_ (*tP*6) structure is antiferromagnetic with magnetic moments of 1.0 µ_B_ on Re atoms. Based on our calculations, the magnetic moment on Re atoms is 2.1 µ_B_, and the band gap is 1.5 eV.

Ir(IV) oxide IrO_2_ crystallizes in a tetragonal crystal structure with space group *P*4_2_/*mnm* (no. 136) [167]. IrO_2_ (*tP*6) is considered to be paramagnetic in the temperature range of 4.2−300 K, and we found an antiferromagnetic spin configuration to be energetically the most favorable at 0 K [152]. The magnetic moments are 0.5 µ_B_. The symmetry of the magnetically ordered structure is lowered to subgroup *Cmmm* (no. 65), identically to VO_2_ (*tP*6) (Figure 3d). The lattice parameters of the calculated structure differ from the experimental data by –0.2% for *a*, *b*, and +0.8% for *c*. IrO_2_ is a metallic conductor [168].

### 2.5. D-Metal Oxides with Molecular Structures

Some binary *d*-metal oxides exist as molecular crystals, where molecular units are held together by weak intermolecular interactions (van der Waals forces): CrO_3_ (*oS*16), MoO_3_ (*oP*16), WO_3_ (*tP*8), Mn_2_O_7_ (*mP*72), Tc_2_O_7_ (*oP*36), RuO_4_ (*cP*40), RuO_4_ (*mS*20), and OsO_4_ (*mS*20). Even though the studied molecular crystals are nonmagnetic, they represent interesting cases because the weak intermolecular interactions are not described properly by standard DFT methods such as PBE or PBE0 [169,170]. Table 2 shows a summary of the optimized lattice parameters for the binary *d*-metal oxides with molecular crystal structures.

While DFT-PBE0/TZVP without dispersion correction results in the overestimation of the lattice parameters, the D3 dispersion correction typically significantly underestimates the lattice parameters. For example, the error in lattice constant *b* of Tc_2_O_7_ −7% for DFT-D3(ZD), compared with +2% without dispersion correction. Most of the studied molecular crystals show ionic bonding, which may be a challenging situation for the DFT-D3 scheme. We also tested the effects of the three-body dispersion term (ABC) on some molecular crystals but found only a negligible effect and no significant improvements.

### 2.6. Mercury Oxides

**Figure 11:**
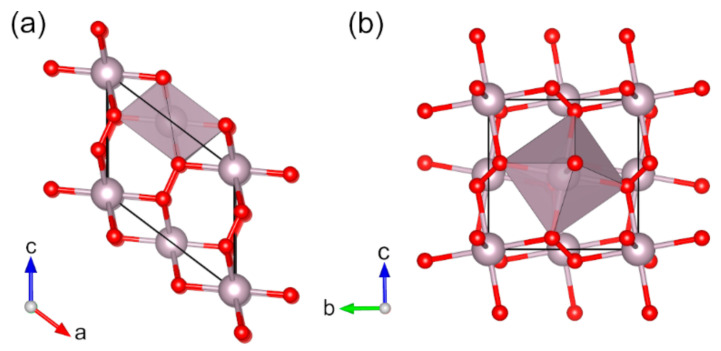
Optimized structures of (**a**) *α*-HgO_2_ (*mS*6) and (**b**) *β*-HgO_2_ (*oP*12) (red: oxygen, almond: mercury). Coordination octahedra of Hg atoms are shown in almond color.

Finally, we discuss in more detail some mercury oxides which are rarely mentioned in the literature and have never been carefully studied: *α*-HgO_2_ (*mS*6) with space group *C*2/*m* (no. 12) and *β*-HgO_2_ (*oP*12) with space group *Pbca* (no. 61) (Figure 11) [179,180,181,182]. The crystal structure of *α*-HgO_2_ has been refined assuming a monoclinic symmetry, yielding a distorted CsCl-type structure. Originally, a rhombohedral unit cell with *α* close to 90° was proposed [181]. *β*-HgO_2_ has been studied more extensively and adopts a distorted version of the cubic MgO_2_ structure of group 12 oxides ZnO_2_ and CdO_2_ [179,180,181].

Based on our calculations, errors in the lattice parameters *a*, *b*, and *c*, compared with experimental data are +35.9%, −25.5%, and 30.5% for *α*-HgO_2_ (*mS*6) and −10.0%, −8.9%, and +13.8% for *β*-HgO_2_ (*oP*12). Such large errors were not observed for any other d-metal oxide included in the study. The errors are not expected to be due to the DFT-PBE0 method or the used basis set, because the other studied mercury oxides, HgO (*oP*8) and HgO (*hP*6), are described well by DFT-PBE0 (the errors in the lattice parameters are less than 1.3%) (see Supplementary Materials). Our findings motivate further experimental studies on the crystal structures of these oxides. For *β*-HgO_2_, our final optimized geometry corresponds to the cubic MgO_2_ structure in space group *Pa*-3 (no. 205). No imaginary vibrational frequencies were observed when a harmonic frequency calculation was carried out in this space group.

## 3. Materials and Methods

All quantum chemical calculations were carried out using the CRYSTAL14 and CRYSTAL17 program packages [40,183]. The structures were fully optimized within the applied space groups by using hybrid PBE0 density functional method (DFT-PBE0, 25% exact exchange) [184,185]. All-electron, Gaussian-type triple-*ζ*-valence + polarization (TZVP) basis sets based on Karlsruhe def2 basis sets were utilized [186]. Scalar relativistic effects were taken into account by means of relativistic effective core potentials for elements Y–Hg. The molecular basis sets were adapted for solid-state calculations, and all basis sets are given as Supplementary Materials. Furthermore, the results obtained with a smaller split-valence + polarization (SVP) basis set are reported in the Supplementary Materials. For some molecular and layered oxides, where weak intermolecular or interlayer interactions could play a role, Grimme’s D3 dispersion correction scheme was tested both with zero-damping and Becke–Johnson damping [41,178,187]. List of the Monkhorst-Pack-type *k*-meshes used for sampling the reciprocal space is given in the Supplementary Materials. Spin-unrestricted formalism was used for all magnetic *d*-metal oxides. Tightened tolerance factors (TOLINTEG) of 8, 8, 8, 8, and 16 were used for the evaluation of the Coulomb and exchange integrals. Default geometry optimization criteria and DFT integration grids of CRYSTAL were used. Harmonic frequency calculations were carried out as implemented in the CRYSTAL software [188,189].

In general, calculations on magnetic oxides were carried out with the following strategy: if experimental data on the magnetic ground state of the crystal structure were available, the reported ground state was calculated. However, there are crystal structures which are only reported as paramagnetic at the room temperature, but the low-temperature magnetic ground state has not been reported. In such cases, we investigated their magnetic and diamagnetic ground states at 0 K, testing various diamagnetic (DM), ferromagnetic (FM), ferrimagnetic (FiM), or antiferromagnetic (AFM) configurations to find the energetically favorable spin configuration (relative energies are given in the Supplementary Materials). We also checked different spin configurations for crystal structures where the magnetic ground state is not known from the experiment. Spin-orbit coupling was not taken into account in the calculations, as spin-orbit coupling is not yet available in the public version of the CRYSTAL code.

All experimental crystal structures were taken from Inorganic Crystal Structure Database (ICSD) [190] or from the Crystallography Open Database (COD) [191,192]. The structures optimized at the DFT-PBE0/TZVP level of theory, including spin configurations for magnetic structures, are available as Supplementary Materials. Structural figures were created using the VESTA software [193].

## 4. Conclusions

We have carried out a comprehensive and systematic computational study of 100 bulk binary *d*-metal oxides by hybrid DFT-PBE0 method. We reported detailed information on the crystal structures including space groups, spin configurations, band gaps, and atomic magnetic moments, which are consistent with the experimental data. For the first time, we found a few problematic cases such as *α*- and *β*-HgO_2_ where crystallographic data, considered to be correct for a long time, seem to be inaccurate. We identified the magnetic ground state of the crystal structures at 0 K, which are known to be paramagnetic. Our study shows that hybrid DFT methods represent a reliable methodology for the description of such strongly correlated systems as *d*-metal oxides. The database facilitates future studies on the more complex properties of the binary *d*-metal oxides and provides a dataset for benchmarking new computational methods.

## Figures and Tables

**Table 1:** Pearson symbols, space groups, magnetic ground states, spin magnetic moments for the metal atoms (µ_B_), and band gaps of the studied magnetic binary *d*-metal oxides.

Oxide	Pearson Symbol ^a^	Space Group of Nonmagnetic Unit Cell	Space Group of Magnetic Unit Cell	Magnetic Ground State ^b^	Magnetic Moment (µ_B_) ^c^		Band Gap (eV)	
					Calc.	Exp.	Calc.	Exp.
3*d* metals								
Ti_2_O_3_	*hR*10	*R*-3*c* (167)	*R*3*c* (161)	AFM	0.9		2.7	0.1 [42]
α-Ti_3_O_5_	*oS*32	*Cmcm* (63)	*Cm* (8)	FiM	1.0		2.0	
β-Ti_3_O_5_	*mS*32	*C*2/*m* (12)	*Cm* (8)	AFM	0.9, 1.0		1.3	0.14 [43]
γ-Ti_3_O_5_	*mS*32	*I*2/*c* (15)	*P*1 (1)	AFM	1.0		2.3	
δ-Ti_3_O_5_	*mS*32	*P*2/*a* (13)	*P*-1 (2)	AFM	1.0		2.4	0.07 [44]
λ-Ti_3_O_5_	*mS*32	*C*2/*m* (12)	*Cm* (8)	AFM	1.0		1.7	
V_2_O_3_	*hR*10	*R*-3*c* (167)	*R*3*c* (161)	AFM/AFM	2.0		3.0	
V_2_O_3_	*mS*20	*I*2/*a* (15)	*P*2/*c* (13)	AFM/AFM	2.0	1.2 [45]	2.8	0.6 [46]
VO_2_	*mP*12	*P*2_1_/*c* (14)	*P*2_1_ (4)	PM/AFM	1.1		3.0	0.6–0.7 [47]
VO_2_	*mS*12	*C*2/*m* (12)	*Cm* (8)	PM/AFM	1.1		3.3	
VO_2_	*tP*6	*P*4_2_/*mnm* (136)	*Cmmm* (65)	PM/AFM	1.1		2.8	-
Cr_2_O_3_	*hR*10	*R*-3*c* (167)	*R*3*c* (161)	AFM/AFM	3.0	ca. 2.7 [48]	5.1	3.2–3.4 [49,50]
CrO_2_	*tP*6	*P*4/*mnm* (136)	*P*4/*mnm* (136)	FM/FM	2.4	2.01 [51]		
MnO	*cF*8	*Fm*-3*m* (225)	*R*-3*m* (166)	AFM/AFM	4.8	4.58 [52]	3.9	3.6–4.2 [53,54]
MnO	*hP*4	*P*6_3_*mc* (186)	*Pmc*2_1_ (26)	AFM	4.8		3.0	
Mn_2_O_3_	*oP*80	*Pbca* (61)	*Pbca* (61)	AFM/AFM	3.9, 4.0	2.3–3.9 [55,56]	3.0	2.17 [57], 2.4 [58]
Mn_2_O_3_	*cI*80	*Ia*-3 (206)	*Ia*-3 (206)	PM/FM	4.1			
Mn_3_O_4_	*tI*28	*I*4_1_/*amd* (141)	*I*4_1_/*amd* (141)	FiM/FiM	3.9, 4.0, 4.9		3.2	1.77–2.72 [59]
MnO_2_	*tI*24	*I*4/*m* (87)	*C*2/*m* (12)	AFM/AFM	3.1		3.4	1.32 [60]
MnO_2_	*oP*12	*Pnam* (62)	*Pmc*2_1_ (26)	AFM/AFM	3.0		3.5	2.57 [61]
MnO_2_	*tP*6	*P*4/*mnm* (136)	*Cmmm (65)*	AFM/AFM	3.1		2.1	0.3 [62]
MnO_2_	*cF*48	*Fd*-3*m* (227)	*Imma* (74)	AFM/AFM	3.1	2.78 [63]	3.7	1.7–3.5 [64]
Fe_3_O_4_	*cF*56	*Fd*-3*m* (227)	*Fd*-3*m* (227)	FiM/FiM	4.0, 4.2	3.82 [65]		
Fe_3_O_4_	*mP*56	*P*2/*c* (13)	*P*2/*c* (13)	FiM/FiM	3.7–4.3	4.17, 4.44 [66]	1.6	0.14 [11]
Fe_2_O_3_	*hR*10	*R*-3*c* (167)	*R*-3 (148)	AFM/AFM	4.2	4.6-5.2 [67]	4.0	5.0 [68,69]
Fe_2_O_3_	*cI*80	*Ia*-3 (206)	I2_1_2_1_2_1_ (24)	AFM/AFM	4.3		3.3	2.2 [70]
Fe_2_O_3_	*oP*40	*Pna*2_1_ (33)	*Pna*2_1_ (33)	FiM/AFM	4.3		4.2	1.6 [71]
CoO	*cF*8	*Fm*3*m* (225)	*R*-3*m* (166)	AFM/AFM	2.7	3.35, 3.8 [72,73]	4.5	4.3 [74]
CoO	*hP*4	*P*6_3_*mc* (186)	*Pmc*2_1_ (26)	AFM	2.8		3.2	
Co_3_O_4_	*cF*56	*Fd-*3*m* (227)	*F*-43*m* (216)	AFM/AFM	2.8	3.88 [75], 3.0 [76]	4.0	0.7 [77]
NiO	*cF*8	*Fm*3*m* (225)	*R*-3*m* (166)	AFM/AFM	1.7	1.64 [78], 1.77 [79], 1.90 [52]	5.2	4.0 [80], 4.3 [81]
CuO	*mS*8	*C*2/*c* (15)	*P*2_1_/*c* (14)	AFM/AFM	0.6	0.65 [82], 0.68 [83,84]	3.8	1.7 [85]
Cu_4_O_3_	*tI*28	*I*4_1_/*amd*	*Imma* (74)	AFM/AFM	0.7	0.66 [86]	2.9	ca. 1.5 [87]
4*d* metals								
MoO_2_	*mP12*	*P*2_1_/*c* (14)	*P*2_1_ (4)	PM/AFM	1.1			
TcO_2_	*mP12*	*P*2_1_/*c* (14)	*P*2_1_ (4)	PM/AFM	2.7		2.4	
RuO_2_	*tP*6	*P*4/*mnm* (136)	*Cmmm (65)*	PM/AFM	1.5	0.05 [88]		
RhO_2_	*tP*6	*P*4_2_/*mnm* (136)	*P*4/*mnm* (136)	PM/FM	0.6			
Ag_3_O_4_	*mP*14	*P*2_1_/*c* (14)	*P*2_1_/*c* (14)	PM/FM	0.2			
5*d* metals								
WO_2_	*mP*12	*P*2_1_/*c* (14)	*P*2_1_ (4)	PM/AFM	0.4			
ReO_2_	*mP*12	*P*2_1_/*c* (14)	*P*2_1_ (4)	PM/AFM	2.1		1.5	
ReO_2_	*oP*12	*Pbcn* (60)	*P*2_1_2_1_2 (18)	PM/AFM	1.1		1.6	
ReO_2_	*tP*6	*P*4_2_/*mnm* (136)	*Cmmm (65)*	AFM	2.7		1.6	
IrO_2_	*tP*6	*P*4_2_/*mnm* (136)	*Cmmm (65)*	PM/AFM	0.5			

^a^ Pearson symbol is used for the description of the crystal structure. It includes the Bravais lattice and the number of atoms in the (nonmagnetic) crystallographic unit cell. ^b^ The ground magnetic state determined in this study (FM: ferromagnetic, AFM: antiferromagnetic, and FiM: ferrimagnetic). In the cases where experimental information on the magnetic ground state is available, the first value is the experimentally determined magnetic ground state, and the second one is the ground state determined here (at 0 K). ^c^ Magnetic moments of the *d*-metal atoms. The calculated values correspond to atomic spin populations.

**Table 2:** Optimized lattice parameters of binary *d*-metal oxides with molecular crystal structures, obtained at the DFT-PBE0/TZVP and DFT-PBE0-D3/TZVP levels of theory. Errors with respect to experimental lattice parameters are shown in parentheses.

Oxide	Pearson Symbol	Space Group	*a* (Å)			*b* (Å)			*c* (Å)		
			No D3	D3 ZD ^a^	D3 BJ ^b^	No D3	D3 ZD	D3 BJ	No D3	D3 ZD	D3 BJ
CrO_3_ [171]	*oS*16	*C*2*cm* (40)	5.748 (0.1%)	5.688 (−1.0%)	5.710 (−0.6%)	8.979 (4.9%)	8.050 (−5.9%)	8.218 (−4.0%)	4.925 (2.8%)	4.715 (−1.6%)	4.711 (−1.6%)
MoO_3_ [172]	*oP*16	*Pbnm* (62)	14.477 (4.5%)	13.380 (−3.5%)	13.515 (−2.5%)	3.695 (0%)	3.697 (0%)	3.692 (−0.1%)	3.972 (0.2%)	3.955 (−0.2%)	3.941 (−0.5%)
WO_3_ [173]	*tP*8	*P*4/*nmm* (129)	5.314 (0.2%)	5.294 (−0.2%)	5.297 (−0.1%)				4.020 (2.2%)	4.018 (2.1%)	4.014 (2.0%)
Mn_2_O_7_ [174]	*mP*72	*P*2_1_/*c* (14)	6.986 (2.8%)	6.693 (−1.5%)	6.697 (−1.4%)	17.504 (4.9%)	16.494 (−1.2%)	16.493 (−1.2%)	9.598 (1.5%)	9.023 (−4.6%)	9.063 (−4.1%)
Tc_2_O_7_ [175]	*oP*36	*Pbca* (61)	13.852 (0.7%)	13.543 (−1.5%)	13.535 (−1.6%)	7.600 (2.2%)	6.908 (−7.1%)	7.033 (−5.5%)	5.762 (2.6%)	5.337 (−5.0%)	5.353 (−4.7%)
RuO_4_ [176]	*cP*40	*P*-43*n* (218)	8.761 (3.0%)	8.254 (−3.0%)	8.359 (−1.8%)						
RuO_4_ [176]	*mS*20	*C*2/*c* (15)	9.562 (2.8%)	9.092 (−2.3%)	9.146 (−1.7%)	4.534 (3.1%)	4.231 (−3.8%)	4.318 −1.8%)	8.673 (2.6%)	8.177 (−3.3%)	8.285 (−2.0%)
OsO_4_ [177]	*mS*20	*C*2/*c* (15)	9.514 (1.4%)	9.066 (−3.3%)	9.058 (−3.4%)	4.572 (1.3%)	4.321 (−4.3%)	4.327 (−4.2%)	8.632 (0%)	8.212 (−4.8%)	8.250 (−4.4%)

^a^ DFT-D3 with zero-damping scheme [41]. ^b^ DFT-D3 with Becke–Johnson damping scheme [178].

## Data Availability

An up-to-date Git repository of the studied *d*-metal oxides is available at https://github.com/aalto-imm/d-oxides (accessed on 30 December 2021).

## Supplementary Materials

The following supporting information can be downloaded online. Table S1: Summary of the studied binary d-metal oxides, Table S2: Lattice parameters of the studied binary d-metal oxides, and Table S3: Energy comparisons of different magnetic configurations for the paramagnetic binary d-metal oxides. All structural data in CIF format, GTO basis sets used in the calculations.
