# Supplementary material for: Structural Properties and Magnetic Ground States of 100 Binary d-Metal Oxides Studied by Hybrid Density Functional Methods
Source: Molecules. 2022 Jan 27;27(3):874. doi: 10.3390/molecules27030874 (PMC8838575; doi:10.3390/molecules27030874)
Supplement: Supplementary file 1 [file molecules-27-00874-s001.zip › MOx_SI.pdf]

Supporting Information for

Structural properties and magnetic ground states of 100 binary *d*-metal oxides studied by hybrid density functional methods

Mikhail S. Kuklin, Kim Eklund, Jarno Linnera, Artturi Ropponen, Nikolas Tolvanen, and Antti J. Karttunen*

Department of Chemistry and Materials Science, Aalto University, 00076 Aalto, Finland. Email: antti.j.karttunen@iki.fi.

Contents

Table S1. Summary of the studied binary *d*-metal oxides.

Table S2. Lattice parameters of the studied binary *d*-metal oxides.

Table S3. Energy comparisons of different magnetic configurations for the paramagnetic binary *d*-metal oxides.

References for Supporting Information

Table S1. Summary of the studied binary *d*-metal oxides.

d-metal	Oxide	ICSD code[a]	Polymorph/Mineral	Space group	Space group for magnetic ground state	Pearson symbol	Oxidation state of the metal	Band gap (DFT-PBE0/TZVP)	Magnetic ground state (DFT-PBE0/TZVP)[d]	*k*-mesh
Sc	Sc_2O_3	26942	Kangite (impure)	*Ia*-3 (206)		*cI*80	III	6.3	DM	4×4×4
Y	Y_2O_3	82420	Yttriaite-(Y)	*Ia*-3 (206)		*cI*80	III	6.5	DM	4×4×4
La	La_2O_3	641600	C-La_2O_3	*Ia*-3 (206)		*cI*80	III	5.8	DM	4×4×4
	La_2O_3	100204	A-La_2O_3	*P*-3*m*1 (164)		*hP*5	III	6.0	DM	12×12×6
Ti	TiO	196273	ε-TiO	*P*-62*m* (189)		*hP*6	II	-	DM	6×6×10[e]
	Ti_2O_3	9646	Tistarite	*R*-3*c* (167)	*R*3*c* (161)	*hR*10	III	2.7	AFM	8×8×8
	TiO_2	202240	Rutile	$P4_2/mnm$ (136)		*tP*6	IV	3.9	DM	8×8×12
	TiO_2	63711	Anatase	$I4_1/amd$ (141)		*tI*12	IV	4.1	DM	8×8×8
	TiO_2	36408	Brookite	*Pbca* (61)		*oP*24	IV	4.4	DM	4×8×8
	Ti_3O_5	50984	α-Ti_3O_5	*Cmcm* (63)	*Cm* (8)	*oS*32	III/IV	2.0	AFM	8×8×4
	Ti_3O_5	26492	β-Ti_3O_5	*C*2/*m* (12)	*Cm* (8)	*mS*32	III/IV	1.3	AFM	8×8×4
	Ti_3O_5	194464	γ-Ti_3O_5	*C*2/*c* (15)	*P*1 (1)	*mS*32	III/IV	2.3	AFM	8×8×6
	Ti_3O_5	194465	δ-Ti_3O_5	*P*2/*a* (13)	*P*-1 (2)	*mS*32	III/IV	2.4	AFM	8×6×4
	Ti_3O_5		λ-Ti_3O_5	*C*2/*m* (12)	*Cm* (8)	*mS*32	III/IV	1.7	AFM	8×8×4
Zr	ZrO_2	82543	Baddeleyite	$P2_1/c$ (14)		*mP*12	IV	5.8	DM	8×8×8
	ZrO_2	23928		$P4_2/nmc$ (137)		*tP*6	IV	6.1	DM	12×12×8
Hf	HfO_2	27313		$P2_1/c$ (14)		*mP*12	IV	6.6	DM	8×8×8
V	V_2O_3	201106	Corundum/Karelianite	*R*-3*c* (167)	*R*3*c* (161)	*hR*10	III	3.0	AFM	8×8×8
	V_2O_3	6286	Karelianite	*C*2/*c* (15)	*P*2/*c* (13)	*mS*20	III	2.8	AFM	8×8×6
	VO_2	74705	Paramontroseite	$P2_1/c$ (14)	$P2_1$ (4)	*mP*12	IV	3.0	AFM	8×8×8
	VO_2	–[b]		*C*2/*m* (12)	*Cm* (8)	*mS*12	IV	3.3	AFM	6×6×6
	VO_2	66665	Paramontroseite	$P4_2/mnm$ (136)	*Cmmm* (65)	*tP*6	IV	2.8	AFM	8×8×12
	V_2O_5	60767	α-V_2O_5/Shcherbinaite, Navajoite (trihydrate)	*Pmmn* (59)		*oP*14	V	4.0	DM	4×12×8
	V_2O_5	80594	γ-V_2O_5	*Pnma* (62)		*oP*28	V	4.1	DM	4×12×4
Nb	NbO	14338		*Pm*-3*m* (221)		*cP*6	II	-	DM	8×8×8
	NbO_2	75197	Distorted rutile	$I4_1/a$ (88)		*tI*96	IV	2.1	DM	4×4×4
	NbO_2	75198	Rutile	$P4_2/mnm$ (136)		*tP*6	IV	-	DM	8×8×12
	Nb_2O_5	71317	*z*-Nb_2O_5	*C*2/*c* (15)		*mS*28	V	4.6	DM	6×6×8
Ta	Ta_2O_5	–	β-Ta_2O_5/Tantite	*Pmna* (53)		*oP*14	V	4.6	DM	6×12×6
Cr	Cr_2O_3	167268	Eskolaite	*R*-3*c* (167)	*R*3*c* (161)	*hR*10	III	5.1	AFM	8×8×8
	CrO_2	202836		$P4_2/mnm$ (136)	$P4_2/mnm$ (136)	*tP*6	IV	-	FM	8×8×12
	CrO_3	16031		*Ama*2 (40)		*oS*16	VI	4.3	DM	8×8×8
Mo	MoO_2	80830	Tugarinovite	$P2_1/c$ (14)	$P2_1$ (4)	*mP*12	IV	-	AFM	8×8×8
	MoO_3	166363	α-MoO_3/Molybdite	*Pnma* (62)		*oP*16	VI	4.2	DM	2×12×12
W	WO_2	8217	Distorted rutile	$P2_1/c$ (14)	$P2_1$ (4)	*mP*12	IV	0.4	AFM	8×8×8
	WO_3	84843	ε-WO_3	*Pc* (7)		*mP*16	VI	3.5	DM	8×8×6
	WO_3	80053	δ-WO_3	*P*-1 (2)		*aP*32	VI	3.4	DM	6×6×6
	WO_3	50727	γ-WO_3/Tungstite (hydride)	$P2_1/c$ (14)		*mP*32	VI	3.3	DM	6×6×6
	WO_3	50729	β-WO_3/Krasnogorite	*Pbcn* (60)		*oP*32	VI	3.3	DM	6×6×6

	WO_3	50732	α-WO_3	*P4/ncc* (130)		*tP*16	VI	2.1	DM	8×8×6
	WO_3	88367		*P4/nmm* (129)		*tP*8	VI	2.1	DM	8×8×8
	WO_3	32001		*P6/mmm* (191)		*hP*12	VI	2.0	DM	6×6×12
Mn	MnO	9864	Manganosite	*Fm*-3*m* (225)	*R*-3*m* (166)	*cF*8	II	3.9	AFM	8×8×8
	MnO	262928		$P6_3mc$ (186)	$Pmc2_1$ (26)	*hP*4	II	3.0	AFM	12×8×8
	Mn_2O_3	24342	Bixbyite	*Pbca* (61)	*Pbca* (61)	*oP*80	III	-	AFM	4×4×4
	Mn_2O_3	9091	Bixbyite	*Ia*-3 (206)	*Ia*-3 (206)	*cI*80	III	-	FM	4×4×4
	Mn_3O_4	109250	Hausmannite	$I4_1/amd$ (141)	*Imma* (74)	*tI*28	II/III	3.2	FiM	6×6×6
	MnO_2	73363	α-MnO_2/Hollandite	*I*4/*m* (87)	*C*2/*m* (12)	*tI*24	IV	3.4	AFM	6×6×4
	MnO_2	78331	γ/R-MnO_2/Ramsdellite	*Pnam* (62)	$Pmc2_1$ (26)	*oP*12	IV	3.5	AFM	12×8×4
	MnO_2	73716	β-MnO_2/Pyrolusite	*P*4/*mnm* (136)	*Cmmm (65)*	*tP*6	IV	2.1	AFM	8×8×12
	MnO_2	193445	λ-MnO_2	*Fd*-3*m* (227)	*Imma* (74)	*cF*48	IV	3.7	AFM	8×8×8
	Mn_2O_7	60821		$P2_1/c$ (14)		*mP*72	VII	3.9	DM	6×2×4 [e]
Tc	TcO_2	173151		$P2_1/c$ (14)	$P2_1$ (4)	*mP*12	IV	2.4	AFM	8×8×8
	Tc_2O_7	16226		*Pbca* (61)		*oP*36	VII	4.9	DM	2×6×8
Re	ReO_2	647349		$P2_1/c$ (14)	$P2_1$ (4)	*mP*12	IV	1.5	AFM	8×8×8
	ReO_2	24060		*Pbcn* (60)	$P2_12_12$ (18)	*oP*12	IV	1.6	AFM	8×8×8
	ReO_2	154021	Rutile	$P4_2/mnm$ (136)	*Cmmm* (65)	*tP*6	IV	1.6	AFM	8×8×12
	ReO_3	77679		*Pm*-3*m* (221)		*cP*4	VI	-	DM	12×12×12
	Re_2O_7	15217		$P2_12_12_1$ (19)		*oP*72	VII	4.8	DM	2×2×8
Fe	Fe_3O_4	85806	Magnetite	*Fd*-3*m* (227)	*Fd*-3*m* (227)	*cF*56	II/III	-	FiM	8×8×8
	Fe_3O_4	98088		*P*2/*c* (13)	*P*2/*c* (13)	*mP*56	II/III	1.6	FiM	8×8×2
	Fe_2O_3	40142	α-Fe_2O_3/Hematite	*R*-3*c* (167)	*R*-3 (148)	*hR*10	III	4.0	AFM	8×8×8
	Fe_2O_3	237290	β-Fe_2O_3	*Ia*-3 (206)	$I2_12_12_1$ (24)	*cI*80	III	3.3	AFM	4×4×4
	Fe_2O_3	415250	ε-Fe_2O_3	$Pna2_1$ (33)	$Pna2_1$ (33)	*oP*40	III	4.0, 3.8	FiM	8×4×4
Ru	RuO_2	15071		$P4_2/mnm$ (136)	*Cmmm* (65)	*tP*6	IV	-	AFM	8×8×12
	RuO_4	415303		*P*-43*n* (218)		*cP*40	VIII	4.2	DM	4×4×4
	RuO_4	415306		*C*2/*c* (15)		*mS*20	VIII	4.1	DM	8×8×4
Os	OsO_2	15070		$P4_2/mnm$ (136)		*tP*6	IV	-	DM	8×8×12
	OsO_4	63		*C*2/*c* (15)		*mS*20	VIII	5.1	DM	8×8×4
Co	CoO	9865		*Fm*3*m* (225)	*R*-3*m* (166)	*cF*8	II	4.5	AFM	8×8×8
	CoO	43458		$P6_3mc$ (186)	$Pmc2_1$ (26)	*hP*4	II	3.2	AFM	12×8×8
	Co_3O_4	63165		*Fd*-3*m* (227)	*F*-43*m* (216)	*cF*56	II/III	4.0	AFM	6×6×6
Rh	Rh_2O_3	108941	Corundum	*R*-3*c* (167)		*hR*10	III	3.2	DM	8×8×8
	Rh_2O_3	9206		*Pbca* (61)		*oP*40	III	3.0	DM	8×8×2 [e]
	RhO_2	28498	Rutile	$P4_2/mnm$ (136)	$P4_2/mnm$ (136)	*tP*6	IV	-	FM	8×8×12
Ir	IrO_2	84577	Rutile	$P4_2/mnm$ (136)	*Cmmm* (65)	*tP*6	IV	-	AFM	8×8×12
Ni	NiO	9866	Bunsenite	*Fm*3*m* (225)	*R*-3*m* (166)	*cF*8	II	5.2	AFM	8×8×8
Pd	PdO	24692	Palladinite	$P4_2/mmc$ (131)		*tP*4	II	1.4	DM	12×12×8
Pt	PtO	– [c]		$P4_2/mmc$ (131)		*tP*4	II	1.4	DM	12×12×8
	Pt_3O_4	43002		*Pm*-3*n* (223)		*cP*14	II/IV	-	DM	8×8×8
	PtO_2	24923	α-PtO	$P6_3mc$ (186)		*hP*6	IV	3.6	DM	12×12×4
	PtO_2	4415	β-PtO	*Pnnm* (58)		*oP*6	IV	2.5	DM	8×8×12
Cu	Cu_2O	52043	Cuprite	*Pn*-3*m* (224)		*cP*6	I	2.3	DM	8×8×8
	CuO	67850	Tenorite	*C*2/*c* (15)	$P2_1/c$ (14)	*mS*8	II	3.8	AFM	6×12×6
	Cu_4O_3	100566	Paramelaconite	$I4_1/amd$ (141)	*Imma* (74)	*tI*28	I/II	2.9	AFM	8×8×8

Ag	Ag_2O	174089		*Pn*-3*m* (224)		*cP*6	I	1.7	DM	8×8×8
	AgO	202055		$I4_1/a$ (88)		*tI*32	I/III	1.5	DM	6×6×6
	Ag_2O_3	1509692		*Fdd2* (43)		*oF*40	III	2.3	DM	8×8×8
	Ag_6O_2	26557		*P*-31*m* (162)		*hP*8	0/I	0.5	DM	8×8×8
	Ag_3O_4	202218		$P2_1/c$ (14)	$P2_1/c$ (14)	*mP*14	II/III	-	FM	12×4×8
Au	Au_2O_3	8014		*Fdd*2 (43)		*oF*40	III	2.9	DM	8×8×8
Zn	ZnO	26170	Zincite	$P6_3mc$ (186)		*hP*4	II	3.6	DM	12×12×8
	ZnO_2	60763		*Pa*-3 (205)		*cP*12	II	5.5	DM	8×8×8
Cd	CdO	29290	Monteponite	*Fm*-3*m* (225)		*cF*8	II	1.4	DM	12×12×12
	CdO_2	36151		*Pa*-3 (205)		*cP*12	II	3.8	DM	8×8×8
Hg	HgO	14124	Montroydite	*Pnma* (62)		*oP*8	II	2.9	DM	6×8×12
	HgO	639125		$P3_221$ (154)		*hP*6	II	2.8	DM	12×12×4
	HgO_2	48214	α-HgO_2	*C*2/*m* (12)		*mS*6	II	2.0	DM	12×12×12
	HgO_2	24774	β-HgO_2	*Pbca* (61)		*oP*12	II	2.0	DM	6×6×8

[a] Code in Inorganic Crystal Structure Database (ICSD)
[b] Code in Crystallography Open Database is 1530870
[c] Code in Crystallography Open Database is 4124669
[d] Diamagnetic (DM), ferromagnetic (FM), ferrimagnetic (FiM), and antiferromagnetic (AFM) spin configurations
[e] TOLINTEG 10 10 10 10 20 was used in the CRYSTAL calculations.

Table S2. Optimized lattice parameters of the studied binary *d*-metal oxides. Differences to experimental values are listed in parentheses.

d-metal	Oxide[a]	Pearson symbol	Lattice parameters[b]											
			DFT-PBE0/SVP						DFT-PBE0/TZVP					
			a (Å)	*b* (Å)	*c* (Å)	α (˚)	β (˚)	γ (˚)	*a* (Å)	*b* (Å)	*c* (Å)	α (˚)	β (˚)	γ (˚)
Sc	Sc_2O_3[1]	*cI*80	9.824<br>(–0.3%)						9.851<br>(0.0%)					
Y	Y_2O_3[2]	*cI*80	10.577<br>(–0.2%)						10.605<br>(0.1%)					
La	La_2O_3[3]	*cI*80	11.430<br>(0.9%)						11.452<br>(1.1%)					
	La_2O_3[4]	*hP*5	3.958<br>(0.5%)		6.177<br>(0.7%)				3.964<br>(0.7%)		6.187<br>(0.8%)			
Ti	TiO[5]	*hP*3	4.944<br>(–1.0%)		2.869<br>(–0.3%)				4.951<br>(–0.9%)		2.872<br>(–0.2%)			
	Ti_2O_3	*hR*10	5.224<br>(1.3%)		13.482<br>(–0.9%)				5.237<br>(1.6%)		13.488<br>(–0.9%)			
	TiO_2[6]	*tP*6	4.571<br>(–0.5%)		2.959<br>(0.0%)				4.584<br>(–0.2%)		2.964<br>(0.2%)			
	TiO_2[7]	*tI*12	3.750<br>(–0.9%)		9.704<br>(2.0%)				3.769<br>(–0.4%)		9.673<br>(1.7%)			
	TiO_2[8]	*oP*24	9.206<br>(0.3%)	5.446<br>(0.0%)	5.102<br>(–0.7%)				9.213<br>(0.4%)	5.454<br>(0.1%)	5.128<br>(–0.2%)			
	α-Ti_3O_5	*oS*32	9.992<br>(1.5%)	3.769<br>(–0.8%)	9.981<br>(–0.1%)		89.9<br>(–0.1%)		10.019<br>(1.8%)	3.776<br>(–0.6%)	10.004<br>(0.2%)		89.9<br>(–0.1%)	
	β-Ti_3O_5	*mS*32	9.942<br>(2.0%)	3.811<br>(0.2%)	9.510<br>(0.7%)		91.8<br>(–0.2%)		10.040<br>(3.0%)	3.825<br>(0.6%)	9.486<br>(0.5%)		91.9<br>(0.0)	
	γ-Ti_3O_5	*mS*32	5.161<br>(1.7%)	5.728<br>(1.2%)	7.037<br>(–2.0%)	109.1<br>(–0.5%)	90.7<br>(0.8%)	116.1<br>(–0.5%)	5.174<br>(2.0%)	5.736<br>(1.4%)	7.057<br>(–1.7%)	109.0<br>(–0.6%)	90.8<br>(0.8%)	116.1<br>(–0.4%)
	δ-Ti_3O_5	*mS*32	5.132<br>(1.4%)	7.087<br>(–1.7%)	10.045<br>(0.8%)	108.5<br>(–0.8%)	90.0<br>(0.0%)	90.0<br>(0.0%)	5.145<br>(1.7%)	7.108<br>(–1.4%)	10.064<br>(1.0%)	108.6<br>(–0.6%)	90.0<br>(0.0%)	90.0<br>(0.0%)
	λ-Ti_3O_5	*mS*32	9.883<br>(0.5%)	3.776<br>(–0.5%)	10.089<br>(1.0%)		89.7<br>(–1.4%)		9.913<br>(0.8%)	3.780<br>(–0.3%)	10.019<br>(1.3%)		89.6<br>(–1.5%)	
Zr	ZrO_2[9]	*mP*12	5.154<br>(0.2%)	5.231<br>(0.4%)	5.302<br>(–0.2%)		99.0<br>(–0.2%)		5.169<br>(0.5%)	5.226<br>(0.3%)	5.327<br>(0.3%)		99.0<br>(–0.2%)	
	ZrO_2[10]	*tP*6	3.599<br>(–1.1%)		5.157<br>(–2.1%)				3.602<br>(–1.0)		5.170<br>(–1.9%)			
Hf	HfO_2[11]	*mP*12	5.175<br>(1.2%)	5.205<br>(0.6%)	5.338<br>(0.8%)		99.1<br>(–0.1%)		5.127<br>(0.3%)	5.235<br>(1.2%)	5.244<br>(–1.0%)		98.4<br>(–0.7%)	
V	V_2O_3	*hR*10	5.046<br>(1.9%)		13.786<br>(–1.5%)				5.053<br>(2.0%)		13.821<br>(–1.3%)			
	V_2O_3	*mS*20	5.499<br>(–0.9%)	5.070<br>(1.4%)	7.295<br>(0.6%)		95.7<br>(–1.1%)		5.509<br>(–0.7%(	5.079<br>(1.5%)	7.309<br>(0.8%)		95.7<br>(–1.0%)	
	VO_2	*mP*12	5.376<br>(0.1%)	4.453<br>(0.0%)	5.357<br>(0.0%)		112.0<br>(0.0%)		5.372<br>(0.0%)	4.457<br>(0.1%)	5.361<br>(0.0%)		112.0<br>(0.1%)	

	VO_2	*mS*12	12.077<br>(0.4%)	3.754<br>(1.6%)	6.332<br>(–1.4%)		106.1<br>(0.0%)		12.094<br>(0.5%)	3.758<br>(1.8%)	6.347<br>(–1.1%)		106.2<br>(0.1%)	
	VO_2	*tP*6	6.232<br>(–3.2%)	6.232<br>(–3.2%)	2.998<br>(5.0%)				6.251<br>(–2.9%)	6.251<br>(–2.9%)	3.001<br>(5.1%)			
	V_2O_5[12]	*oP*14	11.450<br>(–0.5%)	3.546<br>(–0.5%)	4.478<br>(2.5%)				11.455<br>(–0.5%)	3.550<br>(–0.4%)	4.625<br>(5.9%)			
	V_2O_5[13]	*oP*28	10.062<br>(1.2%)	3.563<br>(-0.6%)	10.212<br>(1.7%)				10.115<br>(1.7%)	3.567<br>(–0.5%)	10.503<br>(4.6%)			
Nb	NbO[14]	*cP*6	4.202<br>(–0.2%)						4.209<br>(0.0%)					
	NbO_2[15]	*tI*96	13.787<br>(0.6%)		5.938<br>(–0.8%)				13.791<br>(0.6%)		5.956<br>(–0.5%)			
	NbO_2[15]	*tP*6	4.959<br>(2.3%)		2.869<br>(–5.4%)				4.962<br>(2.4%)		2.878<br>(–5.1%)			
	Nb_2O_5[16]	*mS*28	12.797<br>(0.4%)	4.897<br>(0.3%)	5.599<br>(0.7%)		105.1<br>(0.1%)		12.821<br>(0.6%)	4.911<br>(0.6%)	5.609<br>(0.9%)		104.8<br>(–0.2%)	
Ta	Ta_2O_5[17]	*oP*14	6.448<br>(0.4%)	3.770<br>(0.0%)	7.720<br>(0.2%)				6.441<br>(0.2%)	3.763<br>(–0.2%)	7.691<br>(–0.2%)			
Cr	Cr_2O_3	*hR*10	4.965<br>(0.2%)		13.541<br>(–0.4%)				4.976<br>(0.4%)		13.571<br>(–0.2%)			
	CrO_2	*tP*6	4.359<br>(–1.4%)		2.943<br>(0.9%)				4.368<br>(–1.2%)		2.952<br>(1.2%)			
	CrO_3	*oS*16	5.731<br>(–0.2%)	8.824<br>(3.1%)	4.781<br>(–0.2%)				5.748<br>(0.1%)	8.979<br>(4.9%)	4.925<br>(2.8%)			
Mo	MoO_2	*mP*12	5.552<br>(0.2%)	4.865<br>(0.2%)	5.638<br>(0.5%)		119.4<br>(0.0%)		5.557<br>(0.3%)	4.866<br>(0.2%)	5.660<br>(0.9%)		119.6<br>(0.1%)	
	MoO_3	*oP*16	14.228<br>(2.7%)	3.694<br>(–0.1%)	3.967<br>(0.1%)				14.477<br>(4.5%)	3.695<br>(0.0%)	3.972<br>(0.2%)			
W	WO_2	*mP*12	5.587<br>(0.4%)	4.953<br>(1.2%)	5.581<br>(0.3%)		118.5<br>(–1.6%)		5.554<br>(–0.2%)	4.927<br>(0.6%)	5.585<br>(0.4%)		118.5<br>(–1.6%)	
	WO_3[18]	*mP*16	5.415<br>(2.6%)	5.430<br>(5.3%)	7.771<br>(1.4%)		90.0<br>(–1.9%)		5.416<br>(2.6%)	5.444<br>(5.6%)	7.731<br>(0.9%)		90.4<br>(–1.5%)	
	WO_3[19]	*aP*32	7.520<br>(2.8%)	7.757<br>(3.1%)	7.866<br>(2.3%)	90.0<br>(1.3%)	90.0<br>(–1.0%)	90.0<br>(–1.1%)	7.485<br>(2.4%)	7.751<br>(3.0%)	7.870<br>(2.4%)	90.0<br>(1.3%)	90.0<br>(–1.0%)	90.0<br>(–1.0%)
	WO_3[20]	*mP*32	7.472<br>(2.0%)	7.741<br>(2.3%)	7.900<br>(2.2%)		90.0<br>(–0.5%)		7.437<br>(1.5%)	7.777<br>(2.8%)	7.904<br>(2.3%)		90.0<br>(–0.5%)	
	WO_3[20]	*oP*32	7.472<br>(1.8%)	7.741<br>(2.2%)	7.900<br>(2.0%)				7.439<br>(1.3%)	7.778<br>(2.7%)	7.903<br>(2.0%)			
	WO_3[20]	*tP*16	5.306<br>(0.6%)		8.010<br>(2.1%)				5.310<br>(0.6%)		8.041<br>(2.5%)			
	WO_3	*tP*8	5.341<br>(0.7%)		4.004<br>(1.7%)				5.314<br>(0.2%)		4.020<br>(2.2%)			
	WO_3[21]	*hP*12	7.444<br>(2.0%)		3.821<br>(–2.0%)				7.417<br>(1.6%)		3.809<br>(–2.3%)			
Mn	MnO	*cF*8	3.150<br>(0.2%)		15.162<br>(–1.6%)				3.159<br>(0.5%)		15.191<br>(–1.4%)			

	MnO	*hP*4	3.440 (2.0%)	5.800 (–0.7%)	5.317 (–1.3%)				3.430 (1.7%)	5.786 (–0.9%)	5.392 (0.1%)			
	Mn_2O_3	*oP*80	9.366 (–0.5%)	9.419 (0.0%)	9.469 (0.5%)				9.384 (–0.3%)	9.437 (0.2%)	9.493 (0.7%)			
	Mn_2O_3	*cI*80	9.436 (0.2%)						9.452 (0.4%)					
	Mn_3O_4	*tI*28	5.777 (0.3%)	5.788 (0.5%)	9.409 (–0.5%)				5.785 (0.4%)	5.796 (0.6%)	9.453 (–0.1%)			
	MnO_2	*tI*24	13.709 (–1.2%)	2.862 (0.5%)	9.698 (–1.2%)		135.0 (0.0%)		13.728 (–1.1%)	2.862 (0.5%)	9.705 (–1.1%)		135.0 (0.0%)	
	MnO_2	*oP*12	2.863 (0.5%)	4.508 (1.2%)	9.237 (–0.9%)				2.862 (0.5%)	4.543 (2.0%)	9.211 (–1.2%)			
	MnO_2	*tP*6	6.177 (–0.8%)	6.177 (–0.8%)	2.877 (0.0%)				6.187 (–0.7%)	6.187 (–0.7%)	2.879 (0.1%)			
	MnO_2	*cF*48	5.702 (0.0%)		8.031 (–0.4%)				5.702 (0.0%)		8.028 (–0.4%)			
	Mn_2O_7	*mP*72	6.926 (1.9%)	16.991 (1.8%)	9.434 (–0.2%)		100.5 (0.3%)		6.985 (2.8%)	17.504 (4.9%)	9.598 (1.5%)		100.2 (0.0%)	
Tc	TcO_2	*mP*12	5.561 (1.4%)	4.608 (–3.1%)	5.571 (0.9%)		111.6 (–5.2%)		5.565 (1.5%)	4.611 (–3.0%)	5.575 (1.0%)		111.6 (–5.2%)	
	Tc_2O_7	*oP*36	13.683 (–0.5%)	7.484 (0.6%)	5.694 (1.4%)				13.852 (0.7%)	7.600 (2.2%)	5.762 (2.6%)			
Re	ReO_2	*mP*12	5.576 (–0.1%)	4.872 (1.3%)	5.618 (0.7%)		118.3 (–2.1%)		5.554 (–0.5%)	4.864 (1.1%)	5.606 (0.5%)		118.1 (–2.3%)	
	ReO_2	*oP*12	4.599 (0.0%)	4.866 (1.2%)	5.695 (0.9%)				4.606 (0.1%)	4.836 (0.6%)	5.688 (0.8%)			
	ReO_2	*tP*6	6.548 (–3.5%)	6.548 (–3.5%)	3.207 (14.2%)				6.542 (–3.6%)	6.542 (–3.6%)	3.197 (13.9%)			
	ReO_3[22]	*cP*4	3.765 (0.4%)						3.758 (0.2%)					
	Re_2O_7[23]	*oP*72	12.642 (1.1%)	15.176 (–0.1%)	5.497 (0.9%)				12.693 (1.5%)	15.443 (1.6%)	5.531 (1.5%)			
Fe	Fe_3O_4	*cF*56	8.390 (–0.1%)						8.403 (0.0%)					
	Fe_3O_4	*mP*56	5.986 (0.7%)	5.946 (0.4%)	16.780 (0.0%)		90.0 (–0.3%)		5.983 (0.6%)	5.976 (0.9%)	16.810 (0.2%)		90.0 (–0.3%)	
	Fe_2O_3	*hR*10	5.053 (0.3%)		13.683 (–0.5%)				5.054 (0.4%)		13.725 (–0.2%)			
	Fe_2O_3	*cI*80	9.422 (0.2%)	9.393 (–0.1%)	9.436 (0.3%)				9.422 (0.2%)	9.393 (–0.1%)	9.437 (0.3%)			
	Fe_2O_3	*oP*40	5.095 (0.5%)	8.769 (0.4%)	9.514 (1.0%)				5.100 (0.6%)	8.793 (0.6%)	9.524 (1.1%)			
Ru	RuO_2	*tP*6	6.384 (0.5%)	6.384 (0.5%)	3.118 (0.4%)				6.384 (0.5%)	6.385 (0.5%)	3.122 (0.5%)			
	RuO_4	*cP*40	8.626 (1.4%)						8.761 (3.0%)					

	RuO_4	*mS*20	9.362<br>(0.6%)	4.505<br>(2.5%)	8.542<br>(1.0%)		116.6<br>(–0.2%)		9.562<br>(2.8%)	4.534<br>(3.1%)	8.673<br>(2.6%)		116.5<br>(–0.2%)	
Os	OsO_2[24]	*tP*6	4.479<br>(–0.5%)		3.209<br>(0.8%)				4.474<br>(–0.6%)		3.211<br>(0.9%)			
	OsO_4	*mS*20	9.229<br>(–1.6)	4.476<br>(–0.9%)	8.462<br>(–1.9%)		116.6<br>(0.0%)		9.514<br>(1.4%)	4.572<br>(1.3%)	8.632<br>(0.0%)		116.6<br>(–0.1%)	
Co	CoO	*cF*8	2.959<br>(–1.8%)		14.903<br>(0.9%)				3.021<br>(0.2%)		14.714<br>(–0.4%)			
	CoO	*hP*4	3.278<br>(2.1%)	5.650<br>(1.6%)	5.172<br>(–1.3%)				3.276<br>(2.0%)	5.650<br>(1.6%)	5.222<br>(–0.3%)			
	Co_3O_4	*cF*56	8.079<br>(–0.1%)						8.090<br>(0.1%)					
Rh	Rh_2O_3[25]	*hR*10	5.128<br>(0.0%)		13.903<br>(0.4%)				5.138<br>(0.2%)		13.862<br>(0.1%)			
	Rh_2O_3[26]	*oP*40	5.146<br>(0.0%)	5.454<br>(0.2%)	14.717<br>(0.1%)				5.153<br>(0.1%)	5.454<br>(0.2%)	14.726<br>(0.2%)			
	RhO_2	*tP*6	4.494<br>(0.2%)		3.097<br>(0.3%)				4.495<br>(0.2%)		3.104<br>(0.5%)			
Ir	IrO_2	*tP*6	6.367<br>(–0.1%)	6.367<br>(–0.1%)	3.187<br>(0.9%)				6.360<br>(–0.2%)	6.360<br>(–0.2%)	3.191<br>(1.0%)			
Ni	NiO	*cF*8	2.949<br>(–0.2%)		14.403<br>(–0.5%)				2.963<br>(0.3%)		14.482<br>(0.1%)			
Pd	PdO[27]	*tP*4	3.061<br>(1.0%)		5.327<br>(–0.1%)				3.071<br>(1.4%)		5.317<br>(–0.3%)			
Pt	PtO[28]	*tP*4	3.136<br>(1.9%)		5.284<br>(–1.0%)				3.137<br>(1.9%)		5.273<br>(–1.2%)			
	Pt_3O_4[29]	*cP*14	5.620<br>(0.6%)						5.612<br>(0.5%)					
	PtO_2[30]	*hP*6	3.133<br>(1.1%)		8.703<br>(4.6%)				3.119<br>(0.6%)		9.023<br>(8.4%)			
	PtO_2[31]	*oP*6	4.536<br>(1.1%)	4.524<br>(–0.2%)	3.162<br>(0.8%)				4.544<br>(1.2%)	4.486<br>(–1.0%)	3.158<br>(0.6%)			
Cu	Cu_2O[32]	*cP*6	4.296<br>(0.6%)						4.318<br>(1.1%)					
	CuO	*mS*8	7.638<br>(2.0%)	3.334<br>(–2.8%)	6.401<br>(0.5%)	90.0<br>(0.0%)	94.15<br>(–1.3%)	90.0<br>(0.0%)	7.565<br>(1.1%)	3.423<br>(–0.2%)	6.372<br>(0.1%)	90.0<br>(0.0%)	94.8<br>(–0.7%)	90.0<br>(0.0%)
	Cu_4O_3	*tI*28	5.817<br>(–0.3%)	5.817<br>(–0.3%)	10.028<br>(1.0%)				5.859<br>(0.4%)	5.859<br>(0.4%)	9.977<br>(0.5%)			
Ag	Ag_2O[33]	*cP*6	4.742<br>(0.1%)						4.771<br>(0.7%)					
	AgO[34]	*tI*32	6.864<br>(0.5%)		9.200<br>(0.9%)				6.875<br>(0.6%)		9.209<br>(1.0%)			
	Ag_2O_3[35]	*oF*40	12.848<br>(–0.2%)	10.522<br>(0.3%)	3.728<br>(1.8%)				12.872<br>(0.0%)	10.556<br>(0.6%)	3.713<br>(1.3%)			
	Ag_6O_2[36]	*hP*8	5.325<br>(0.1%)		4.945<br>(–0.1%)				5.356<br>(0.7%)		4.925<br>(–0.5%)			

	Ag_3O_4	*mP*14	3.621<br>(1.2%)	9.255<br>(0.5%)	5.709<br>(0.6%)		106.9<br>(0.7%)		3.621<br>(1.2%)	9.255<br>(0.5%)	5.709<br>(0.6%)		106.9<br>(0.7%)	
Au	Au_2O_3[37]	*oF*40	10.642<br>(1.2%)	12.917<br>(0.4%)	3.883<br>(1.2%)				10.611<br>(0.9%)	12.923<br>(0.4%)	3.858<br>(0.5%)			
Zn	ZnO[38]	*hP*4	3.256<br>(0.2%)		5.183<br>(–0.5%)				3.267<br>(0.5%)		5.207<br>(0.0%)			
	ZnO_2[39]	*cP*12	4.872<br>(0.0%)						4.894<br>(0.5%)					
Cd	CdO[40]	*cF*8	4.691<br>(–0.1%)						4.688<br>(–0.1%)					
	CdO_2[41]	*cP*12	5.298<br>(–0.3%)						5.301<br>(–0.2%)					
Hg	HgO[42]	*oP*8	6.676<br>(1.0%)	5.300<br>(–4.0%)	3.462<br>(–1.7%)				6.702<br>(1.3%)	5.536<br>(0.3%)	3.551<br>(0.8%)			
	HgO[43]	*hP*6	3.515<br>(–1.6%)		8.566<br>(–1.2%)				3.610<br>(1.1%)		8.704<br>(0.4%)			
	HgO_2	*mS*6	4.571<br>(2.3%)	4.734<br>(–13.3%)	4.070<br>(15.7%)		111.7<br>(3.0%)		6.076<br>(35.9%)	4.066<br>(-25.5)	4.592<br>(30.5%)		127.1<br>(17.2%)	
	HgO_2	*oP*12	5.445<br>(–10.4%)	5.449<br>(–9.3%)	5.434<br>(13.2%)				5.471<br>(–10.0%)	5.472<br>(–9.0%)	5.465<br>(13.8%)			

[a] Literature citations are given here for the oxides that are not discussed in detail in the main paper.

[b] Non-magnetic experimental lattice parameters have been transformed so that they correspond to the unit cell used in the spin-polarized calculations. Differences to the experimental values are reported for the magnetic unit cell.

Table S3. Energy comparisons of different magnetic configurations for the paramagnetic binary *d*-metal oxides (DFT-PBE0/SVP level of theory).

Oxide	Pearson symbol	Magnetic configuration[b]	Relative energy (kJ mol^{-1} per atom)
Ti_2O_3[a]	*hR*10	DM	6.8
		FM	12.4
		**AFM**	**0.0**
α-Ti_3O_5[a]	*oS*32	DM	9.0
		FM	2.0
		FiM	2.3
		**AFM**	**0.0**
γ-Ti_3O_5[a]	*mS*32	DM	9.7
		FM	3.7
		FiM	3.4
		**AFM**	**0.0**
δ-Ti_3O_5[a]	*mS*32	DM	8.7
		FM	0.4
		FiM	0.1
		**AFM**	**0.0**
VO_2	*mP*12	DM	14.9
		FM	14.0
		**AFM**	**0.0**
VO_2	*mS*12	DM	26.4
		FM	7.0
		**AFM**	**0.0**
VO_2	*tP*6	DM	28.4
		FM	18.8
		**AFM**	**0.0**
NbO_2	*tP*6	**DM**	**0.0**
		FM	1.8
		AFM	0.0
	*tI*96	**DM**	**0.0**
		FM	5.9
		AFM	8.3
MoO_2	*mP*12	DM	3.5
		FM	3.7
		**AFM**	**0.0**
Mn_2O_3	*cI*80	DM	132.5
		**FM**	**0.0**
		FiM	2.2
TcO_2	*mP*12	DM	28.1
		FM	28.2
		**AFM**	**0.0**
ReO_2	*mP*12	DM	16.5
		FM	38.8
		**AFM**	**0.0**
ReO_2	*oP*12	DM	7.4
		FM	8.6
		**AFM**	**0.0**
ReO_2	*tP*6	DM	25.0
		FM	5.1
		**AFM**	**0.0**
RuO_2	*tP*6	DM	11.5
		FM	16.5
		**AFM**	**0.0**
RhO_2	*tP*6	DM	11.6
		**FM**	**0.0**
		AFM	1.9
IrO_2	*tP*6	DM	7.2
		**AFM**	**0.0**
		FM	0.1
Ag_3O_4	*mP*14	DM	2.8
		**FM**	**0.0**
		FiM	2.7
		AFM	2.7

[a] Magnetic ground state of the structure is not known from the experiment.
[b] Diamagnetic (DM), ferromagnetic (FM), ferrimagnetic (FiM), or antiferromagnetic (AFM) configuration.

References

(1) Knop, O.; Hartley, J. M. Refinement of the Crystal Structure of Scandium Oxide. *Can. J. Chem.* **1968**, *46*, 1446–1450.
(2) Maslen, E. N.; Streltsov, V. A.; Ishi. A Synchrotron X-Ray Study of the Electron Density in C-Type Rare Earth Oxides. *Acta Cryst.* **1996**, *B52*, 414–422.
(3) Felsche, J. A New Form of La2O3. *Kurze Orig.* **1969**, 212.
(4) Aldebert, P.; Traverse, J. P. Etude Par Diffraction Neutronique Des Structures De Haute Temperature De La2O3 et Nd2O3. *Mat. Res. Bull.* **1979**, *14*, 303–323.
(5) Amano, S.; Bogdanovski, D.; Yamane, H.; Terauchi, M.; Dronskowski, R. ε-TiO, a Novel Stable Polymorph of Titanium Monoxide. *Angew. Chemie - Int. Ed.* **2016**, *55* (5), 1652–1657.
(6) Burdett, J. K.; Hughbanks, T.; Miller, G. J.; Smith, J. V.; Richardson, J. W. Structural-Electronic Relationships in Inorganic Solids: Powder Neutron Diffraction Studies of the Rutile and Anatase Polymorphs of Titanium Dioxide at 15 and 295 K. *J. Am. Chem. Soc.* **1987**, *109* (12), 3639–3646.
(7) Howard, C. J.; Sabine, T. M.; Dickson, F. Structural and Thermal Parameters for Rutile and Anatase. *Acta Crystallogr. Sect. B* **1991**, *47* (4), 462–468.
(8) Meagher, E. P.; Lager, G. A. Polyhedral Thermal Expansion in the TiO2 Polymorphs: Refinement of the Crystal Structures of Rutile and Brookite at High Temperature. *Can. Mineral.* **1979**, *17*, 77–85.
(9) Gualtieri, A.; Norby, P. Rietveld Refinement Using Synchrotron X-Ray Powder Diffraction Data Collected in Transmission Geometry Using an Imaging-Plate Detector: Application to Standard M-. *J. Appl. Crystallogr.* **1996**, *29*, 707–713.
(10) Teufer, G. The Crystal Structure of Tetragonal ZrO2. *Acta Crystallogr.* **1962**, *15* (11), 1187.
(11) Ruh, R.; Corfield, P. W. R. Crystal Structure of Monoclinic Hafnia and Comparison with Monoclinic Zirconia. *J. Am. Ceram. Soc.* **1970**, *53* (3), 126–129.
(12) Enjalbert, R.; Galy, J. A Refinement of the Structure of V2O5. *Acta Cryst.* **1986**, *C42*, 1467–1469.
(13) Cocciantelli, J. M.; Gravereau, P.; Doumerc, J. P.; Puchard, M.; Hagenmuller, P. On the Preparation and Characterization of a New Polymorph of V2O5. *J. Solid State Chem.* **1991**, *93*, 497–502.
(14) Bowman, A. L.; Wallace, T. C.; Yarnell, J. L.; Wenzel, R. G. The Crystal Structure of Niobium Monoxide. *Acta Crystallogr.* **1966**, *21* (5), 843–843.
(15) Bolzan, A. A.; Fong, C.; Kennedy, B. J. A Powder Neutron Diffraction Study of Semiconducting and Metallic Niobium Dioxide. *J. Solid State Chem.* **1994**, *113*, 9–14.
(16) Ercit, S. Verfeinerung Der Struktur Des ζ-Nb2O5 Und Deren Beziehungen Zu Den Strukturen Des Rutils Und Thoreauliths. *Mineral. Petrol.* **1991**, *43*, 217–223.
(17) Hollerweger, R.; Holec, D.; Paulitsch, J.; Bartosik, M.; Daniel, R.; Rachbauer, R.; Polcik, P.; Keckes, J.; Krywka, C.; Euchner, H.; et al. Complementary Ab Initio and X-Ray Nanodiffraction Studies of Ta2O5. *Acta Mater.* **2015**, *83*, 276–284.
(18) Woodward, P. M.; Sleight, A. W.; Vogt, T. Ferroelectric Tungsten Trioxide. *J. Solid State Chem.* **1997**, *131* (1), 9–17.
(19) Woodward, P. M.; Sleight, A. W.; Vogt, T. Structure Refinement of Triclinic Tungsten Trioxide. *J. Phys. Chem. Solids* **1995**, *56* (10), 1305–1315.
(20) Vogt, T.; Woodward, P. M.; Hunter, B. A. The High-Temperature Phases of WO3. *J. Solid State Chem.* **1999**, *144* (1), 209–215.
(21) Gerand, B.; Nowogrocki, G.; Guenot, J.; Figlarz, M. Structural Study of a New Hexagonal Form of Tungsten Trioxide. *J. Solid State Chem.* **1979**, *29*, 429–434.
(22) Jorgensen, J. E.; Jorgensen, J.; Batlogg, B.; Remeika, J.; Axe, J. Order Parameter and Critical Exponent for the Pressure-Induced Phase Transitions in ReO3. *Phys. Rev. B* **1986**, *33* (7), 4793–4798.
(23) Krebs, B.; Muller, A.; Beyer, H. H. Crystal Structure of Rhenium (7) Oxide. *Inorg. Chem.* **1969**, *8* (3), 436–443.
(24) Boman, C.-E.; Danielsen, J.; Haaland, A.; Jerslev, B.; Schäffer, C. E.; Sunde, E.; Sørensen, N. A. Precision Determination of the Crystal Structure of Osmium Dioxide. *Acta Chemica Scandinavica*. 1970, pp 123–128.
(25) Coey, J. M. D. The Crystal Structure of Rh_2O_3. *Acta Crystallogr. Sect. B Struct. Crystallogr. Cryst. Chem.* **1970**, *26* (11), 1876–1877.
(26) Biestterbos, J. W. M.; Hornstra, J. The Crystal Structure of the High-Temperature, Low-Pressure Foem of Rh2O3. *J. Less-Common Met.* **1973**, *30*, 121–125.
(27) Waser, J.; Levy, H. A.; Peterson, S. W. The Structure of PdO. *Acta Crystallogr.* **1953**, *6* (7), 661–663.
(28) Moore, W. J.; Pauling, L. The Crystal Structures of the Tetragonal Monoxides of Lead, Tin, Palladium, and Platinum. *J. Am. Chem. Soc.* **1941**, *63* (5), 1392–1394.
(29) Muller, O.; Roy, R. Formation and Stability of the Platinum and Rhodium Oxides at High Oxygen Pressures and the Structures of Pt3O4,b-PtO2 and RhO2. *Journals Less-Common Met.* **1968**, *16*, 129–146.
(30) Hoekstra, H. R.; Siegel, S.; Gallagher, F. x. *Reaction of Platinum Dioxide with Some Metal Oxides*; American Chemical Society: Washington, DC, 1971.
(31) Siegel, S.; Hoekstra, H. R.; Tani, B. S. The Crystal Structure of Beta-Platinum Dioxide. *J. Inorg. Nucl. Chem.* **1969**, *31*, 3803–3807.
(32) Restori, R.; Schwarzenbach, D. Charge Density in Cuprite , Cu20. *Acta Crystallogr. Sect. B Struct. Crystallogr. Cryst. Chem.* **1986**, *B42*, 201–208.
(33) Srivastava, S. P.; Srivastava, R. C.; Pandey, S. D. Thermal Expansion of Ag2O. *J. Phys. Chem. C* **1977**, *43*, 1463–1464.
(34) Yvon, K.; Bezinge, A. Structure and Magnetic Properties of Tetragonal Silver(I,III) Oxide, AgO. *J. Solid State Chem.* **1986**, *65*, 225–230.
(35) Standke, B.; Jansen, M. Ag2O3, a Novel Binary Oxide. *Angew. Chem. Intl. Ed. Engl.* **1985**, *24*, 118–119.
(36) Beesk, W.; Jones, P. G.; Rumpel, H.; Schwarzmann, E.; Sheldrick, G. M. X-Ray Crystal Structure of Ag6O2. *J.C.S. Chem. Comm.* **1981**, *166*, 664–665.
(37) Jones, P. G.; Rumpel, H.; Schwarzmann, E.; Sheldrick, G. M.; Paulus, H. Gold (Ill) Oxide. *Acta Cryst.* **1979**, *B35*, 1435–1437.
(38) Abrahams, S. C.; Bernstein, J. L. Remeasurement of the Structure of Hexagonal ZnO. *Acta Crystallogr. Sect. B Struct. Crystallogr. Cryst. Chem.* **1969**, *25* (7), 1233–1236.
(39) Puselj, M.; Ban, Z.; Morvaj, J. On the Peroxides of Zinc and Cadmium. *Croat. Chem. Acta* **1985**, *58* (3), 283–288.
(40) Cimino, A.; Marezio, M. Lattice Parameter and Defect Structure of Cadmium Oxide Containing Foreign Atoms. *J. Phys. Chem. Solids* **1960**, *17*, 57–64.
(41) Hoffman, C. W. W.; Ropp, R. C.; Mooney, R. W. Preparation, Properties and Structure of Cadmium Peroxide. *J. Am. Chem. Soc.* **1959**, *81* (15), 3830–3834.
(42) Aurivillius, K. Least-Squares Refinement of the Crystal Structures of Orthorhombic HgO and of Hg2O2NaI. *Acta Chem. Scand.* **1964**, *18*, 1305–1306.
(43) Laruelle, P. Sur Le Polymorphisme de l'oxyde Mercurique. *Compt. Rend.* **1955**, *241*, 802–805.
